# Integrating palliative care into respiratory care for people with advanced COPD: study protocol for the international EU PAL-COPD stepped wedge cluster randomised controlled trial of the ICLEAR-EU intervention

**DOI:** 10.1136/bmjresp-2026-004150

**Published:** 2026-11-02

**Authors:** Michelle Martine Huub Van Der Meer, Julie Stevens, Frederick Daenen, Nancy Preston, Paul Marsden, Anne Wichmann, Kim Beernaert, Laure Dombrecht, Ágnes Csikós, Mogens Grønvold, Nanna Maria Hammer, Peter May, Diogo Queiroz Almeida, Sandra Martins Pereira, Pablo Hernández-Marrero, Els Verschuur, Libby Sallnow, Luc Deliens, Koen Pardon, consortium EU PAL-COPD, Mishka Beutels, Yvonne Engels, Manon Wubbels, Kim Eecloo, Marie Van de Walle, Catherine Walshe, Paul Marsden, Tim Gatheral, Didar Karadag Akkaya, Elham Nikram, Maria Conceição Silva, Eva Poszgai, Balázs Daniel Fülöp, Réka Gajdócsi, André Ribeiro

**Affiliations:** 1 End-of-Life Care Research Group, Vrije Universiteit Brussel, Brussels, Belgium; 2 Lancaster University, Lancaster, UK; 3 Manchester University NHS Foundation Trust, Manchester, UK; 4 Radboud University Medical Center, Nijmegen, Netherlands; 5 End-of-Life Care Research Group, Ghent University, Ghent, Belgium; 6 Pecsi Tudomanyegyetem - University of Pécs, Pécs, Hungary; 7 Kobenhavns Universitet, Copenhagen, Denmark; 8 King’s College London, London, UK; 9 Universidade Catolica Portuguesa, Porto, Portugal; 10 Universidade dos Açores | Fundação Gaspar Frutuoso (FGF), Ponta Delgada, Portugal; 11 Long Alliantie Nederland, Amersfoort, Netherlands; 12 University College London, London, UK

**Keywords:** Palliative Care, Pulmonary Disease, Chronic Obstructive, COPD Exacerbations

## Abstract

**Introduction:**

Individuals with advanced chronic obstructive pulmonary disease (COPD) often experience significant symptom burden, unmet care needs and frequent hospitalisations, yet remain underserved by palliative care. Evidence suggests palliative care can improve quality of life and reduce avoidable hospital readmissions but robust multi-country randomised evaluations are lacking. Building on a successful pilot subsequently adapted, this protocol presents the EU PAL-COPD study evaluating the ICLEAR-EU intervention to reduce potentially avoidable respiratory-related readmission within 90 days and improve quality of life for individuals with advanced COPD and their families across European health systems.

**Methods and analysis:**

A stepped-wedge cluster randomised controlled trial evaluating the effectiveness of the ICLEAR-EU intervention in Belgium, the UK, the Netherlands, Hungary, Denmark and Portugal, comparing ICLEAR-EU to usual care. ICLEAR-EU is an integrated non-pharmacological service intervention for hospitalised patients with advanced COPD to identify and address palliative care needs. Hospitals (clusters) cross over from usual care to intervention in four 6-month wedges after a 30-day transition/training period. Eligible participants are community-dwelling adults with advanced COPD admitted ≥48 hours for an acute exacerbation; each may nominate a family caregiver. We aim to enrol 1224 patients across 18 hospitals (three per country). The primary endpoint is the percentage of patients with respiratory-related hospital readmissions within 90 days of baseline (or until death if within 90 days). Secondary endpoints include patient and family caregiver quality of life, well-being, healthcare utilisation and cost-effectiveness at 30- and 90-day follow-up. Process evaluation will be conducted throughout. Analyses follow intention-to-treat using mixed-effects models accounting for clustering, time and country. The statistical analysis plan is provided as supplementary material.

**Ethics and dissemination:**

Ethics approval was obtained in all participating countries and hospitals. Findings on effectiveness, cost-effectiveness and process evaluation will be disseminated via academic channels and established project communication channels.

**Trial registration number:**

ISRCTN45800298.

**WHAT IS ALREADY KNOWN ON THIS TOPIC**

- Individuals with advanced chronic obstructive pulmonary disease (COPD) often experience a substantial symptom burden, unmet care needs and frequent hospitalisations, yet remain underserved by palliative care. Existing research indicates that palliative care can improve quality of life and reduce avoidable hospital readmissions.

**WHAT THIS STUDY ADDS**

- The innovative EU PAL-COPD trial is the first cross-national stepped wedge cluster randomised controlled trial of a service-level intervention integrating palliative care into routine care for individuals with advanced COPD, targeting hospitalised patients and their family caregivers.

**HOW THIS STUDY MIGHT AFFECT RESEARCH, PRACTICE OR POLICY**

- The comprehensive evaluation of the ICLEAR-EU intervention, with impact measured in terms of intervention effectiveness, cost-effectiveness and a process and implementation evaluation, will potentially offer clinicians, researchers and policy makers a well-tested and evaluated service intervention that can be transferred to wider hospital settings.

## Introduction

Chronic obstructive pulmonary disease (COPD), a treatable but incurable respiratory condition, is the fourth leading cause of death worldwide.^1^ Individuals with COPD experience high symptom burden, including progressive breathlessness,^2^ fatigue, anxiety and depression,^3^ along with social and existential needs.^4 5^ In advanced stages, quality of life (QoL) declines and exacerbations become frequent, leading to repeated hospitalisations^2 6^ and accelerating decline in physical functioning and QoL. Readmissions carry a high risk: around 11% 1-year mortality and over 30% 90-day readmissions post-discharge.^7^ They are also costly, with COPD accounting for more than half of European respiratory healthcare expenditure.^6^

Palliative care reduces hospital readmissions^8^ and improves QoL by addressing physical, psychological, social and spiritual and existential needs.^5 9^ Despite comparable needs to individuals with advanced cancer, individuals with COPD remain underserved in referral and access to specialist palliative care.^10–13^ When they do receive such care, it is usually only in the final weeks of life.^2 12–15^ This results in limited capacity for optimised symptom management and shared-decision making, shifting to acute clinical deterioration management.^13^ Furthermore, the frequency and quality of palliative care and end-of-life care conversations are generally poor.^16^ To address this, international guidance supports integrating palliative care, including advance care planning, into routine respiratory care for persons with advanced or end-stage COPD.^13 17–19^ This supports individuals with COPD and their families in making choices about future care,^20^ and strengthening care continuity, resulting in improved patient outcomes and healthcare system efficiency.^21 22^ Despite guideline recommendations, models integrating palliative care approaches into COPD management remain rare, and evidence from high-quality multi-country trials is lacking. This highlights the need to evaluate proactive, feasible and sustainable integrated palliative care models for routine COPD care.

The EU PAL-COPD project addresses this gap by testing the innovative, non-pharmacological service-based ICLEAR-EU intervention. The intervention aims to reduce potentially avoidable respiratory-related readmissions within 90 days, achieve better QoL and improve well-being for individuals with advanced COPD and their family caregivers. It focuses on five key components: (1) early identification of palliative care needs, (2) structured communication about goals of care and sharing them with the patient, family and care team, (3) shared decision-making on levels of escalation, (4) initiating advance care planning (ACP) and (5) ongoing review and management of palliative care needs. The intervention builds on a UK-based proof-of-concept study 20 23 and other research showing promising effects, including reduced psychosocial impact of COPD and healthcare utilisation, increased home deaths and ACP choices, and improved communication.^20 23 24^

This protocol describes the international EU PAL-COPD stepped wedge cluster-randomised controlled trial, evaluating the effectiveness and cost-effectiveness of the ICLEAR-EU intervention compared with usual care in six European countries. The primary aim is to test whether the ICLEAR-EU intervention reduces respiratory-related readmissions within 90 days and improves QoL and well-being for individuals with advanced COPD and their families by integrating palliative care into routine respiratory care. A concurrent process evaluation will evaluate implementation and integration feasibility across usual care in European settings, exploring contextual barriers and facilitators for effective and sustainable implementation, and mechanisms driving outcomes as perceived by patients, family caregivers and care professionals.

## Methods and analysis

This protocol is reported following the Standard Protocol Items: Recommendations for Interventional Trials 2025 statement.^25^ A structured overview of the study design and methods is provided in online supplemental file 1.^26^

10.1136/bmjresp-2026-004150.supp1 Supplementary data

### Trial design

A stepped wedge cluster-randomised controlled trial (SWC-RCT)^27^ will be used to compare the ICLEAR-EU intervention with treatment as usual. Clustering and randomisation are at hospital level. Each hospital will go through four wedges with a duration of 6 months for a total of 24 months, followed by a final 90-day follow-up of recruited participants. Before cross-over from control to intervention, a 30-day transition period will be included for clinician training and support. Control and intervention populations are discrete. A schematic representation is shown in figure 1.

**Figure 1:**
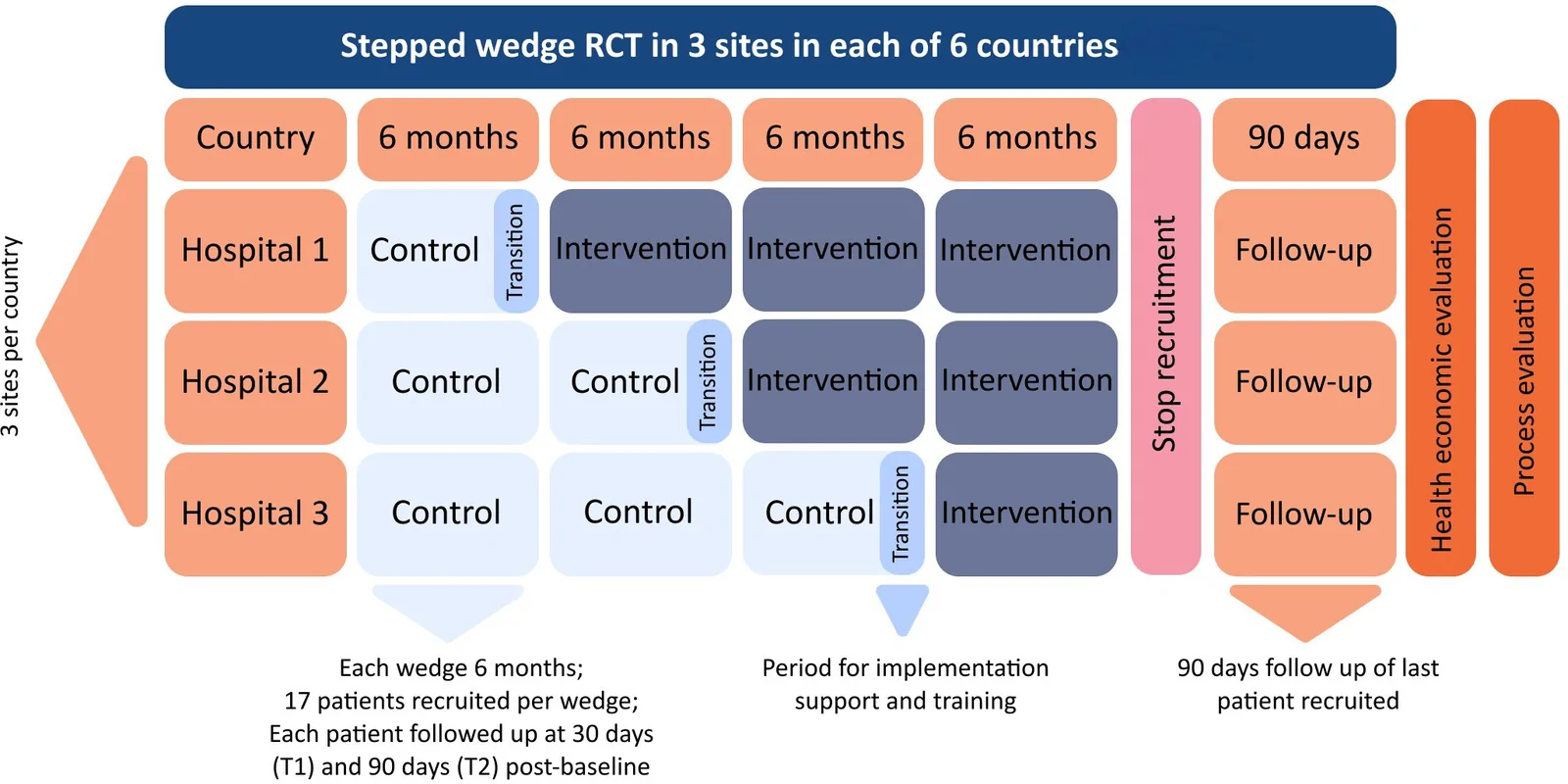
The design of the EU PAL-COPD stepped wedge cluster-randomised controlled trial (RCT), showing the design for one country with three hospitals. Each of the six participating countries will follow this design. The sequence of cross-over from control to intervention is randomised. The recruitment period covers a total of 24 months, with a 90-day follow-up of the last patient recruited after recruitment stops. Effectiveness, health economic and process evaluation data are collected throughout the trial.

### Trial setting

The SWC-RCT will proceed similarly in 18 hospitals across six countries (Belgium, the Netherlands, the UK, Denmark, Hungary and Portugal). Each country will include three hospitals, typically admitting a minimum of 100–500 patients annually for COPD-related causes, having inpatient respiratory beds and indicating willingness to implement the ICLEAR-EU intervention. Community/primary care are involved through clinical team communication and review of ICLEAR-EU documentation during follow-up consultations. Data will be collected from patients in the hospital and at home after discharge.

### Study population

Patients and family caregivers must meet the inclusion criteria in table 1. Patients may be enrolled with or without a family caregiver, defined as ‘any relative, friend, or partner who has a significant relationship and provides assistance (physical, social, and/or psychological)’^28^ to the patient.

**Table 1:** Patient and family caregiver inclusion and exclusion criteria

Patient	Family caregiver
Inclusion criteria	
Have a diagnosis of advanced COPD* Admission to the respiratory ward of the hospital that lasts ≥48 hours (or likely to be admitted for ≥48 hours) for an acute exacerbation^†^ Live at home	Identified by the patient as the person who gives him or her the most help and support at home on a regular basis Age 18 years or over
Exclusion criteria	
Currently receiving care from a formally recognised specialised palliative care team Cognitive impairment preventing informed consent as judged by the treating respiratory physician and by the researchers. In case of doubts, the researcher will consult the corresponding treating respiratory physician Not able to speak or understand the language in which measurements are conducted, these being: English Dutch Danish Portuguese Hungarian Patients can be included in the study only once and cannot be re-enrolled during the overall duration of the study, even if at a different wedge	Cognitive impairment preventing informed consent as judged by the treating respiratory physician and by the researchers. In case of doubts, the researcher will consult the corresponding treating respiratory physician Not able to speak or understand the language in which measurements are conducted
*Advanced COPD 1. Spirometry (FEV_1_): Severe COPD: 30%≤FEV_1_<50% predicted **OR** Very severe COPD: FEV_1_<30% predicted OR 2. High symptom burden: Modified Medical Research Council (mMRC) >2 **OR** COPD Assessment Test (CAT) >20 OR 3. High-risk exacerbation history: ≥1 exacerbation leading to previous hospitalisation in the past year **OR** ≥1 exacerbation leading to previous ICU admission in the past year **^†^Acute exacerbation** Event characterised by dyspnoea/cough and/or cough/sputum worsening over <14 days.^19^ Individuals may have other significant comorbidities (including pneumonia, heart failure), but the predominant reason for admission will be due to COPD in the opinion of the investigator	

COPD, chronic obstructive pulmonary disease; FEV_1_, forced expiratory volume in 1 s; ICU, intensive care unit.

Eligible clinicians must provide care to individuals with advanced COPD and participate in the ICLEAR-EU intervention.

### Recruitment

In each country, three eligible hospitals have been identified. Hospitals received preparatory information about the study and staff duties. Core staff members were identified by the country research teams prior to trial initiation. Core disciplines delivering the ICLEAR-EU intervention are pulmonologists, palliative care physicians and respiratory and palliative care nurses.

Patient and family caregiver recruitment will continue throughout the trial (24 months), using consecutive sampling, with no recruitment during the transition month starting from the moment the training is delivered. Hospitalised patients with COPD exacerbation will be flagged by hospital staff. A respiratory clinician will approach eligible patients concerning study participation, providing study details and an introduction letter. Through eligible patients, family caregivers will be identified. If present at the information visit, they receive information simultaneously and are invited to participate. If absent, patients will be asked to identify an eligible family caregiver and permit research team contact. With consent, contact details are shared with the research team, who will obtain patient and family caregiver written informed consent. This process is supported by a short explanatory video.

The research team will inform clinicians about process evaluation procedures before informed consent is sought, prior to data collection, no later than the transition phase.

Participants are informed they can withdraw without justification at any time and without consequences. If provided, reasons for withdrawal are documented.

### Sample size calculation

Sample size calculation follows the approach by Hussey and Hughes.^29^ We specify a minimum clinically important difference of 15% in the number of patients readmitted to the hospital within 90 days after baseline. Based on existing literature^30^ and research team expertise, we estimate 35% readmissions at baseline. We use a conservative estimate of the intracluster correlation coefficient of 0.05 and apply a correction for 30% dropout.

Sample size calculations yield an average of 17 patients recruited per hospital per wedge (68 patients total per hospital, 204 per country, 1224 patients in total). Over-recruitment up to 25 participants per wedge is allowed to account for loss during the hospital transition phase. This gives at least 90.0% power to detect a difference of 15% at two-sided α=0.05.

### Randomisation

Study sites will be allocated using stratified randomisation at hospital level, with stratification by the hospital’s country. Hospitals will be randomly assigned to one of three implementation timepoints (IT1, IT2, IT3), with each timepoint including exactly six hospitals, one per country. Prior to trial initiation, the trial statistician generated the randomisation sequence using R V.4.3.1. The randomisation list and code are securely stored on a Ghent University drive, accessible only by the trial statistician, and encrypted and password-protected throughout the trial.

### Blinding

Hospital time-point allocation will be concealed from research teams and hospital staff at trial onset, until required for intervention implementation. The trial statistician will unblind allocation to the project coordinator at months 4, 10 and 16, who will then unblind the country research teams, which will immediately notify the hospitals. Blinding of hospital staff, patients and family caregivers is not possible due to the nature of the trial.

### Control

During control wedges, hospitals provide patients with treatment as usual per national healthcare practices and international guidelines. To reduce contamination, patients who already receive care from a formally recognised palliative care team will not be eligible for participation (table 1). Beyond this, there are no restrictions on elements of palliative care permitted at the hospital during the control phase.

### Transition

The transition phase, 30 days before intervention start-up, consists of implementation support and clinicians’ training. The training includes identification, assessing needs and delivering a palliative care approach to patients with advanced COPD, including how to implement the core components of the ICLEAR-EU intervention. Each hospital will identify a ‘clinical champion’ who promotes the intervention and provides respiratory team leadership, enhancing adherence and team commitment. A ‘coordinator’ will also be identified, undertaking tasks such as planning ICLEAR-EU weekly team meetings.

### Intervention

The ICLEAR-EU intervention is a novel, staged, multi-faceted service-level systematic approach to care for those with advanced COPD, addressing palliative needs.

The goal is to identify palliative care needs, facilitate discussions with patients, families and care professionals, prepare an agreed care plan, and integrate palliative care into present and future care strategies. The intervention consists of five core components:

- *Identification* of palliative care needs using the Edmonton Symptom Assessment Scale-Revised (ESAS-r) with three additional questions assessing social and existential needs, or using a local assessment tool alongside standard clinical assessment. Tools must assess needs across physical, psychological, social and existential domains. Belgium, Hungary, Portugal and the UK use the ESAS-r with three additional questions. In Denmark, PRO-Pall is used; this patient-reported outcome measure has previously been used in Danish COPD populations.^31^ In the Netherlands, the USD-4D is used, which is based on the ESAS and validated for Dutch palliative care settings.^32 33^ These tools were permitted as their current use facilitates implementation into practice. The identified needs inform weekly multidisciplinary team discussions between respiratory and palliative care teams, ideally including community and/or primary care.
- *Communication* about goals of care and sharing them with patients, family and care team. The ICLEAR-EU-form documents discussion outcomes between clinicians, patients and families.
- Shared decision-making regarding *Levels of Escalation*. During communication with the patient, family and clinical team, care preferences and levels of escalation are established, documented on the ICLEAR-EU-form and inform the patient management plan for future care.
- Initiating *ACP* conversations, which are conducted with the patient and ideally their family caregiver, led by a member of the clinical team in the hospital or community.
- Ongoing *Review* and management of palliative care needs during follow-up visits with healthcare professionals, and revision of the patient management plan if or when necessary. At least one follow-up visit will be scheduled as part of the intervention.

Participants can express involvement preferences in treatment and care decisions throughout the intervention. The intervention flow is illustrated in figure 2.

**Figure 2:**
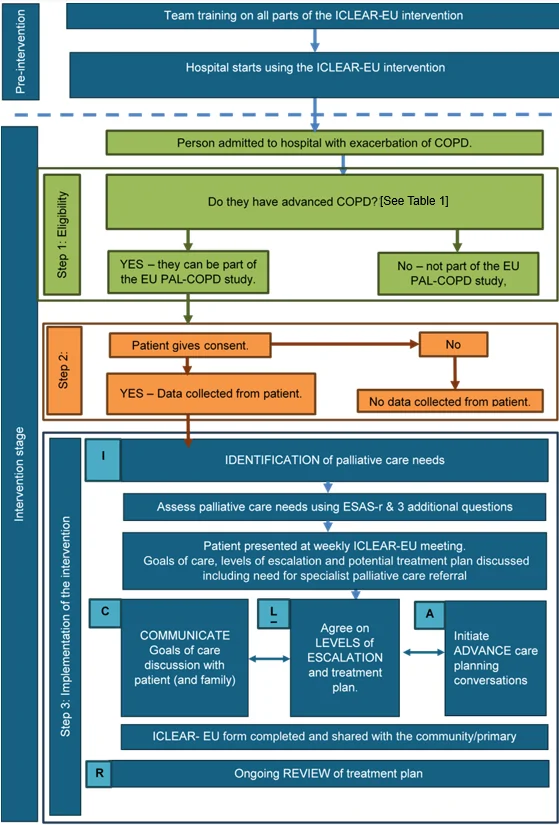
The flow of participants in the ICLEAR-EU intervention. Preintervention preparation is at the hospital/team level via training and implementation start. Patients must meet the study eligibility criteria and give consent for trial participation and data collection before they receive the intervention. The intervention begins with identification of palliative care needs, which are discussed at the weekly ICLEAR-EU meeting. Communication about goals of care, agreement on levels of escalation and a treatment plan and advance care planning take place with the patient (and family), are documented in the ICLEAR-EU form, and regularly reviewed. COPD, chronic obstructive pulmonary disease; ESAS-r, Edmonton Symptom Assessment Scale-Revised.

Clinicians delivering the intervention will be provided with an ICLEAR-EU intervention manual detailing all components, which will be publicly available on CORDIS-EU.

Additional intervention training will be provided on a 6-month basis or as needed, for example, after staff turnover.

### Outcomes

#### Demographics

Participant demographic data will be collected and reported via questionnaires at baseline (online supplemental file 2). The items were, where feasible, drawn from the previous European multicentre DIAdIC study.^34^

10.1136/bmjresp-2026-004150.supp2 Supplementary data

#### Primary outcome

The primary outcome is the percentage of patients with advanced COPD who have a respiratory-related readmission to the hospital within 90 days of baseline (or until death if within these 90 days). This aligns with outcomes in other European studies, allowing comparison.^35^ We hypothesise that fewer individuals in the intervention phase will require hospital readmission.

#### Secondary outcomes

Secondary outcomes were selected to minimise response burden in seriously ill patients while ensuring sufficiently comprehensive and informative data collection. Differences between control and intervention will be measured at 30 and 90 days post-baseline.

The secondary outcomes and data collection timing are shown in table 2.

**Table 2:** Constructs measured in the study, corresponding instruments, scheduling

Construct	Data collection measure	Completed by	Timing		
			**T0 (baseline**)	**T1 (30 days post-baseline**)	**T2 (90 days post-baseline**)
Patient outcomes (collected via questionnaires)					
Perception of illness	Brief IPQ^48^	Patient	x	x	x
Quality of life	SF-CRQ^49^ EQ-5D-5L^50^ ICECAP-SCM^51^	Patient	x	x	x
Mental well-being	PHQ-4^52^	Patient	x	x	x
Existential well-being	MQOL-R existential subscale^53^	Patient	x	x	x
Preferred place of death+whether this has been discussed with healthcare professionals	Questionnaire item	Patient	x	x	x
[Bereaved]caregiver outcomes (collected via questionnaires)					
Quality of life	EQ-5D-5L^50^	Family caregiver	x	x	x
Mental well-being	PHQ-4^52^	Family caregiver	x	x	x
Existential well-being	MQOL-R existential subscale^53^	Family caregiver	x	x	x
Family carer burden	ZBI-12^54^	Family caregiver	x	x	x
Bereaved caregiver views of quality of care and death	VOICES-SF^55^	Bereaved caregiver	3 months post-bereavement		
Healthcare utilisation of enrolled patients					
Place of death	Medical notes or phone general practitioner (GP)	Clinician/researcher	As appropriate		
Concordance between preferred place of death and actual place of death	Questionnaire item medical notes or phone GP	Clinician/researcher	As appropriate		
All-cause mortality	Medical notes	Clinician/researcher	As appropriate		
Number of readmissions to hospital	Medical notes	Clinician/researcher			x
Median length of hospital stays on readmission	Medical notes	Clinician/researcher			x
Number of referrals to specialist palliative care	ICLEAR-EU form and medical notes	Clinician/researcher			x
Intensive care unit (ICU) admissions	Medical notes	Clinician/researcher			x
Emergency department admissions	Medical notes	Clinician/researcher			x
Presence of advance decisions to refuse treatment (ADRTs) and advance care plans (ACPs)	ICLEAR-EU form and medical notes	Clinician/researcher			x
Healthcare utilisation and unpaid family caregiving	CSRI^56^	Patient	x	x	x

Brief IPQ, The Brief Illness Perception Questionnaire; CSRI, Client Service Receipt Inventory; EQ-5D-5L, EuroQoL 5-Dimension 5-level; ICECAP-SCM, The ICECAP Supportive Care Measure; MQOL-R, The McGill Quality of Life Questionnaire–Revised; PHQ-4, Patient Health Questionnaire–4 items; SF-CRQ, The Short-Form Chronic Respiratory Disease Questionnaire; VOICES-SF, Views of Informal Carers–Evaluation of Services Short form; ZBI-12, Zarit Burden Interview–12 items.

#### Health economic evaluation

We will use hospital data to quantify length and intensity of inpatient hospital stays, with intensity determined by admission type. We will collect additional formal healthcare utilisation and unpaid family care using an adapted Client Service Receipt Inventory (CSRI), and estimate costs by combining reported frequencies with nation-specific unit costs. We will collect health-related QoL using EuroQoL 5-Dimension 5-level (EQ-5D-5L), converting responses to quality-adjusted life years (QALYs) using nation-specific population preference weights and mortality data from the main trial. See table 2 for the data collection time points.

#### Process evaluation

An embedded process evaluation will be conducted at all sites, as described below.

#### Current care delivery

To understand hospital care practices underlying the trial and intervention, the ICLEAR-EU coordinator or clinical champion will complete a questionnaire four times per hospital, during the last month of each wedge. If this coincides with transition, it will be completed before the training. This questionnaire will capture different characteristics in palliative care practices during the control and intervention conditions, between hospitals and between countries, as well as changes in practice over time, such as those introduced by new local or national policies.

To assess current practices related to the ICLEAR-EU intervention, a one-time interview with the ICLEAR-EU coordinator or clinical champion will be conducted during transition, before training, following the current care delivery questionnaire to minimise bias.

#### PRISM/RE-AIM

We will use the PRISM/RE-AIM framework^36 37^ to evaluate Reach, Effectiveness, Adoption, Implementation and Maintenance of the intervention alongside key contextual factors. These will be qualitatively and quantitatively assessed, see online supplemental file 3.

10.1136/bmjresp-2026-004150.supp3 Supplementary data

### Data collection and management

#### Data collection

Patient healthcare data (table 2) will be collected by a clinician or researcher through a data retrieval form. If needed, home or community care will be contacted to complete the form. This addresses anticipated increased attrition with disease progression, minimising missing primary outcome data. With patient consent, medical record data collection is continued after study withdrawal.

For secondary outcomes, a data collector will collect the baseline questionnaire (T0, immediately after consent) in-hospital, or if discharged, at home or another convenient location. Follow-up questionnaires will be completed at 30 (T1) and 90 (T2) days post-baseline, with support from a data collector as needed via phone, video, home visit or another convenient location to promote retention and meet participant preferences. If a patient dies during follow-up, participating family caregivers will complete the VOICES-SF questionnaire 3 months post-bereavement. All questionnaires are administered in English or each country’s official language, using existing validated translations, or forward-backward translations of validated English-language instruments. All questionnaires will be completed in a pen-and-paper format.

Cost-effectiveness data (CSRI and EQ-5D-5L) are embedded in the patient and family caregiver questionnaires. Unit costs will be identified by literature search and, where necessary, by calculation by the research team. The economic evaluation will also use the healthcare utilisation data to model intensity of hospital stay for a given patient profile.

For the process evaluation, quantitative and qualitative data will be collected. Per hospital, semi-structured interviews will be conducted:

- During intervention, two patients and two family caregivers will be selected by convenience sampling. Four weeks after hospital discharge, patients will be interviewed about their care experiences. Individual interviews are recommended, though patients may opt for a joint interview with their family caregiver. Three months post-bereavement, two bereaved family caregivers will be interviewed about the care received.
- After the final intervention wedge, two clinicians (coordinator and/or clinical champion) will be interviewed about intervention experiences and maintenance suggestions.

Missing data, discontinued data collection or discontinued participation will be documented by the data collector or researcher in REDCap (Research Electronic Data Capture), alongside the reason, if provided. REDCap is an internationally recognised system for data management and recording for clinical trials. 38 39

Validated data collection forms are cited in table 2 and online supplemental file 3. Self-developed data collection forms are available from the project coordinator on request.

#### Data management

All data will be entered into REDCap, in compliance with Regulation (EU) 2016/679 of the European Parliament and of the Council of 27 April 2016 on the protection of natural persons with regard to the processing of personal data and on the free movement of such data, and repealing Directive 95/46/EC (General Data Protection Regulation, GDPR). REDCap access is limited to research team members. The coordinating team will regularly check data quality and monitor missing data patterns. Data sharing agreements (joint controller agreement) will be put in place, allowing data transfer for research collaboration and analysis within the consortium. A data management plan has been created and will be maintained throughout the project.

At project level, each research partner will store data according to institutional data management rules and applicable legal, ethical and safety provisions of the individual states and EU.

All data will be stored for a minimum of 10 and a maximum of 25 years.

### Statistical methods

All analyses will follow intention-to-treat with a 5% significance level. 95% CIs will be reported where appropriate. All included participants, with either observed or imputed outcome data, will be included in primary and secondary outcome analyses and the health economic evaluation. Participants will be assessed based on entry during a control wedge or intervention wedge. Missing values will be replaced according to a chained equations multiple imputation method under the missing at random assumption. Data not measured because of death are not considered missing. A detailed statistical analysis plan (SAP) is provided in online supplemental file 4. The sections below summarise the main statistical considerations.

10.1136/bmjresp-2026-004150.supp4 Supplementary data

#### Primary outcome

The primary outcome will be compared between control and intervention participants using a logistic mixed model approach. Effect sizes will be expressed as ORs. A random effect will be included for hospital, and fixed effects for condition (control or intervention), country and time. A fixed-effect interaction between country and time will allow varying secular trends.^40^ Covariates age, gender, comfort of living on current income, cohabitation status and hospital characteristics are included as fixed effects, improving estimated intervention effect precision.

#### Secondary outcomes and subgroup comparisons

Secondary outcomes with (at most) one outcome per participant will be analysed using models similar to the primary outcome, with the appropriate link function selected. For repeated measures, an appropriate mixed model will be defined, based on the primary outcome model. We will add the baseline measure and time (categorical) as predictors alongside a treatment-time interaction. Patient-specific random intercepts will be included.

#### Health economic evaluation

Prior to conducting primary cost-effectiveness analysis, we will examine: (a) baseline differences in characteristics associated with outcome and where necessary control for baseline variables; and (b) skewness, kurtosis and heteroscedasticity in cost data and fit an appropriate model. We will address missing cost and outcome data using multiple imputation (MI), ensuring consistency of MI approach with the clinical trial analyses to the greatest extent possible. Recognising the uncertainty associated with the trial design, we will employ a stratified two-stage non-parametric bootstrap resampling procedure for clustered data. We will model costs and outcomes using multi-level models to cluster by country. We will combine trial results with data from other sources to model effects for a lifetime horizon. We will express results in incremental cost-effectiveness ratios (cost per QALY), and cost-effectiveness acceptability curves (probability of cost-effectiveness for different willingness-to-pay thresholds). The primary cost-effectiveness analysis will be undertaken from the healthcare system perspective in each country, with a planned secondary analysis adopting a societal perspective.

#### Process evaluation

Quantitative data will be analysed descriptively and using inferential statistics depending on data type and analysis rationale.

Open coding of qualitative data will be done in English in each country locally. A common codebook will be developed by RUMC. Codes will be grouped into categories and themes in English during a meeting. All research partners will contribute to the analyses and interpretation.

#### Subgroup and sensitivity analyses

Information on subgroup and sensitivity analyses are detailed in the SAP (online supplemental file 4).

### Patient involvement

Patients were actively involved in trial design and will be consulted throughout its conduct. They provided feedback on data collection procedures in the pilot phase. During protocol development, the Trial Steering Committee (TSC), including a patient representative, reviewed the protocol and informed the final version. Following publication of results, participants will be informed via the EU PAL-COPD website, newsletter and social media for a non-specialist audience.

### Confidentiality

All data from participants will be handled in coded fashion and pseudonymised before analysis. No identifiable data will be transferred between partners.

### Trial safety and monitoring

The trial coordinator, Vrije Universiteit Brussel, is responsible for project coordination, risk assessment and contingency planning, quality management, legal and ethics oversight with UCPH.

#### Trial safety

A Data and Safety Monitoring Board (DSMB), comprising three independent experts, safeguards participant privacy and data security. It makes recommendations about trial continuation, modification or stopping to the TSC chair and project coordinator based on 6 monthly reports generated by the trial statistician and data management team.

A standard operating procedure applies to all sites for reporting (serious) adverse events ((S)AEs). They will be recorded in REDCap, and reported to country PIs, local ethics committees, project coordinator, DSMB and TSC. SAEs require expedited reporting.

Based on experience with previous similar studies^23 41 42^ we do not anticipate SAEs related to intervention or trial procedures. Death will not be classified as SAE, as it is an anticipated outcome for some participants. No formal interim analyses or stopping rules are prespecified. Trial participants are referred to existing resources for ancillary and post-trial care, including continued intervention integration where appropriate.

#### Trial monitoring

A TSC oversees the trial, protocol adherence, and monitors progress during 6 monthly, or if needed ad hoc, meetings. It comprises VUB PIs, core trial staff, three independent experts, a patient representative and observers invited as needed.

In each country, a trial manager or PI will oversee trial conduct, implementation, data collection and (S)AEs. An independent trial monitor (ITM) will perform 6 monthly evaluations regarding trial conduct, data quality, and Good Clinical Practice, and report to country PI and VUB team.

### Dissemination policy

Results will be disseminated through peer-reviewed publication, conferences, scientific workshops, lay summaries via the European Respiratory Society newsletter, a final project symposium and the EU PAL-COPD project channels.

Datasets containing personal data will be shared with third parties only on reasonable request, in accordance with informed consent, confidentiality and unilateral data sharing agreements, and local Data Protection Officer guidance, in compliance with GDPR. Open data and research outputs will be freely available from repositories.

## Discussion

Individuals with advanced COPD suffer from significant symptom burden associated with poor QoL and frequent hospital admissions.^2 6^ Palliative care can decrease hospital readmissions and improve QoL.^8 9^ Individuals with advanced COPD have palliative care needs similar to those with (lung) cancer, but are underserved,^10–13^ highlighting need for improvement. Accordingly, we will evaluate effectiveness, cost-effectiveness and implementation of the ICLEAR-EU intervention, an innovative palliative care intervention integrated into routine respiratory care, targeting individuals hospitalised with advanced COPD across European healthcare systems.

To our knowledge, only a few palliative care interventions for individuals with advanced COPD target hospitalised patients.^20 43^ However, they are at high risk of exacerbations, morbidity and mortality,^7^ making hospitalisation a crucial opportunity for intervention. Additionally, previous studies were conducted in single-country settings. One used a pragmatic cluster-controlled design, but randomisation was not possible.^20^ Therefore, this trial is highly innovative as the first cross-national SWC-RCT targeting hospitalised patients with advanced COPD. Also, as most individuals with COPD prefer to be cared for^44^ and die at home,^45^ preventing potentially avoidable respiratory-related hospital readmissions is essential. Research suggests personal care plans reduce hospitalisations^46 47^ and increase home deaths.^47^ The ICLEAR-EU intervention is therefore promising, as it provides participants with a systematically reviewed patient management plan.

This study has several strengths. Evaluation of ICLEAR-EU, including effectiveness, cost-effectiveness and process and implementation outcomes, supports adaptation across European healthcare systems. The multi-centre cross-national design allows transferability to broader hospital settings, and the primary outcome aligns with other European studies, allowing comparison.^35 46^ Moreover, the SWC-RCT is a pragmatic design enabling implementation and effectiveness evaluation. It enhances comparability as each cohort contributes data during both control and intervention wedges. It is also more efficient than parallel cluster designs, maximising within-cluster comparisons over time, typically requiring fewer clusters to obtain adequate statistical power. Additionally, all participating hospitals receive the intervention.^27^ The implementation frameworks RE-AIM^36^ and PRISM^37^ support outcome effects interpretation. Finally, the intervention is based on a UK care model, previously evaluated in a proof-of-concept study showing promising effects.^23^

This study also has potential limitations. Maintaining hospitals’ engagement throughout the 2-year trial, especially those receiving the intervention in the final wedge, may prove challenging. To mitigate this, hospitals will be closely monitored with 6-monthly meetings, and each country will recruit a back-up hospital in case of withdrawal. Also, the ICLEAR-EU intervention has been pilot tested to maximise acceptability and feasibility. Patient-level attrition is expected due to their (declining) health. To reduce burden, questionnaires were pilot tested and adapted where needed and trained data collectors will support questionnaire completion at participants’ preferred location. The study design also has potential limitations. First, selection bias may occur as blinding is not possible. This will be mitigated through consecutive recruitment. Second, contamination may occur during the transition phase after clinicians receive training and apply new knowledge in routine care. Third, temporal contamination may occur when a 90-day follow-up for control patients extends into an intervention wedge. Fourth, the stepped wedge design may involve time-varying intervention effects. These risks are addressed in the SAP. Additionally, patients not consenting during transition or intervention phases may indirectly benefit from the intervention. The intervention, indirect effects, and potential contamination are systematised in online supplemental file 5. Finally, while extensive process evaluation data collection will occur, the study does not record consultations with patients and thus does not assess the quality of communication directly. However, patients and relatives will be able to share their impressions of quality of communication during the interviews as part of the process evaluation. The VOICES questionnaire administered to bereaved caregivers will also be used to assess perceived quality of communication.

10.1136/bmjresp-2026-004150.supp5 Supplementary data

## Data Availability

No data are available. As this manuscript details the study protocol, no datasets were generated/analysed.
