# Supplementary material for: Integrating palliative care into respiratory care for people with advanced COPD: study protocol for the international EU PAL-COPD stepped wedge cluster randomised controlled trial of the ICLEAR-EU intervention
Source: BMJ Open Respir Res. 2026 Nov 2;13(1):e004150. doi: 10.1136/bmjresp-2026-004150 (PMC13735246; doi:10.1136/bmjresp-2026-004150)
Supplement: Supplementary file 1 [file bmjresp-13-1-s001.pdf]

Supplementary Table 1. Administrative information on the EU PAL-COPD stepped wedge cluster randomised controlled trial of the ICLEAR-EU intervention.

Title	Integrating palliative care into respiratory care for individuals with advanced COPD
Primary registry and trial identifying number	International Standard Randomised Controlled Trial Number (ISRCTN)<br><br>ISRCTN45800298<br><br>https://www.isrctn.com/ISRCTN45800298
Date of registration in primary registry	28 October 2024
Secondary identifying numbers	101136621
Source(s) of monetary or material support	European Union under the Horizon Europe Programme (HORIZON-HLTH-2023-DISEASE-03)<br>Innovate UK<br>Swiss State Secretariat for Education Research and Innovation<br>Ministry of Culture and Innovation of Hungary from the National Research, Development and Innovation Fund
Primary sponsor	Vrije Universiteit Brussel (VUB)
Secondary sponsor(s)	NA
Contact for public queries	Prof. dr. Koen Pardon<br>Phone: (+32 0)24774757<br>Email: koen.pardon@vub.be<br><br>Prof. dr. Luc Deliens<br>Phone: (+32 0)24774757<br>Email: luc.deliens@vub.be<br><br>Dr. Julie Stevens<br>Phone: (+32 0)24774757<br>Email: julie.stevens@vub.be
Contact for scientific queries	Prof. dr. Koen Pardon<br>Phone: (+32 0)24774757<br>Email: koen.pardon@vub.be<br><br>Prof. dr. Luc Deliens<br>Phone: (+32 0)24774757<br>Email: luc.deliens@vub.be<br><br>Dr. Julie Stevens<br>Phone: (+32 0)24774757<br>Email: julie.stevens@vub.be

Public title	The ICLEAR service model to integrate palliative care and respiratory medicine for chronic obstructive pulmonary disease patients
Scientific title	A stepped wedge randomised controlled trial and process evaluation of the ICLEAR-EU intervention to integrate palliative care in the treatment of people with advanced COPD and their family caregivers
Countries of recruitment	Belgium<br>United Kingdom<br>The Netherlands<br>Hungary<br>Denmark<br>Portugal
Health condition(s)	Advanced chronic obstructive pulmonary disease (COPD)
Intervention(s)	The ICLEAR-EU intervention: a service-level non-pharmacological intervention based on the principles of integrated palliative care, initiated during hospitalization for persons with advanced COPD, with a focus on communication, determining the level of escalation, making care plans and regular active review.
Key inclusion and exclusion criteria	**INCLUSION CRITERIA:**<br>**Patients:**<br>• Having a diagnosis of advanced COPD:<br>1. Spirometry (FEV1):<br>a. Severe COPD: 30% ≤ FEV1 < 50% predicted OR<br>b. Very severe COPD: FEV1 < 30% predicted<br>OR<br>2. High symptom burden:<br>a. Modified Medical Research Council (mMRC) mMRC > 2 OR<br>b. COPD Assessment Test (CAT) CAT > 20<br>OR<br>3. High risk exacerbation history:<br>a. ≥ 1 exacerbation leading to previous hospitalization in the past year OR<br>b. ≥ 1 exacerbation leading to previous ICU admission in the past year<br>• Admission to the respiratory ward of the hospital that lasts ≥ 48 hours (or likely to be admitted for ≥ 48 hours) for an acute exacerbation<br>• Living at home<br>**Family caregivers:**<br>• Identified by the patient as the person who gives him or her the most help and support at home on a regular basis<br>• Age 18 years or over

	**EXCLUSION CRITERIA:**<br>**Patients:**<br>• Currently receiving care from a formally recognized specialized palliative care team<br>• Cognitive impairment preventing informed consent as judged by treating respiratory physician and by the researchers. In case of doubts, the researcher will consult the corresponding treating respiratory physician.<br>• Not speaking or understanding the language in which measurements are conducted<br>• After first enrolment, patients cannot be re-enrolled during the study<br>**Family caregivers:**<br>• Cognitive impairment preventing informed consent as judged by treating respiratory physician and by the researchers. In case of doubts, the researcher will consult the corresponding treating respiratory physician.<br>• Not speaking or understanding the language in which measurements are conducted
Study type	Type of study: interventional<br>Method of allocation: randomised controlled trial<br>Intervention model: stepped wedge<br>Allocation concealment: blinded (researchers and hospital staff) until 2 months prior to cross-over from control to intervention wedge.<br>Phase: NA
Date of first enrolment	1 September 2025
Sample size	1224 patients in total<br>68 patients per hospital (3 hospitals in each country)
Recruitment status	Recruiting
Primary outcome(s)	The percentage of patients with respiratory-related hospital readmissions within 90 days of baseline (or until death, if within 90 days).
Key secondary outcomes	At 30 days (T1) and 90 days (T2) post-baseline:<br>**Patients**<br>• Perception of illness<br>• Quality of life<br>• Mental wellbeing<br>• Existential wellbeing<br>• Preferred place of death<br>• Presence of advance decisions to refuse treatment and documentation of advance care planning decisions

	**Family caregivers**<br>• Quality of life<br>• Mental wellbeing<br>• Existential wellbeing<br>• Family carer burden<br>• Views on quality of care and death (bereaved caregivers only)<br>**Healthcare utilisation of enrolled patients**<br>• Place of death<br>• Concordance between preferred and actual place of death<br>• All-cause mortality<br>• Number of readmissions<br>• Median length of hospital stay on readmission<br>• Number of hospital days<br>• Number of referrals to specialist palliative care<br>• Number of intensive care unit (ICU) admissions<br>• Number of emergency department admissions<br><br>**Cost-effectiveness**<br>• Patient quality-adjusted life years (primary endpoint)<br><br>**Process evaluation**<br>• PRISM/RE-AIM (context, reach, effectiveness, adoption, implementation and maintenance)
Ethics review	**Belgium**<br>• Ethics committee or Institutional Board: Medical Ethics Committee (MEC) of Ghent University (UGent) and Ghent University Hospital (UZ Gent)/Commissie voor Medische Ethiek U(Z) Gent<br>• Reference number: ONZ-2025-0050<br>• Status: Approved<br>• Approval date: 26 May 2025<br>• Contact: ethisch.comite@uzgent.be<br>**United Kingdom**<br>• Ethics committee or Institutional Board: HRA and Health and Care Research Wales (HCRW)<br>• Reference number: 25/SC/0194<br>• Status: Approved<br>• Approval date: 11 July 2025<br>• Contact: healthandcareresearch@wales.nhs.uk<br>**The Netherlands**

	• Ethics committee or Institutional Board: Medical Ethics Evaluation Commission (METC Oost-Nederland)<br>• Reference number: 2025-17958<br>• Status: The Medical Research Ethics Committee Oost-Nederland concluded this study was not subject to the Medical Research Involving Human Subjects Act (2025-17958).<br>• (Approval) date: 11 March 2025<br>• Contact: metc@amsterdamumc.nl<br>**Hungary**<br>• Ethics committee or Institutional Board: ETT TUKEB – Scientific and Research Ethics Committee of the Medical Research Council, Hungary<br>• Reference number: BM/16149-1/2025<br>• Status: Approved<br>• Approval date: 04 July 2025<br>• Contact: ettv@bm.gov.hu<br>**Denmark**<br>• Ethics committee or Institutional Board: Regional Health Research Ethics Committees in the Capital Region in Denmark<br>• Reference number: F-24036330<br>• Status: NA. The Regional Health Research Ethics Committees in the Capital Region in Denmark waived the need for ethical approval of the project.<br>• Approval date: NA<br>• Contact: vek@regionh.dk<br>**Portugal**<br>• Status and approval dates:<br>• Ethics committee or Institutional Board: 1) Clinical Ethics Committee (CEC) and 2) Board of Administrators (BA) of Hospital do Santo Espírito da Ilha Terceira, Angra do Heroísmo, Terceira Island, Autonomous Region of the Azores, Portugal<br>o Reference number: Nº 10/2025; SAI-HSEIT/2025/1259<br>o Approval dates: 1) 03 September 2025 (CEC) and 2) 11 September 2025 (BA)<br>o Contact: Sandra.MS.Lobao@azores.gov.pt<br>• Ethics committee or Institutional Board: 1) Ethics Committee (CETICA) and 2) Board of

	Administrators (BA) of Hospital da Horta, Horta and Madalena, Faial and Pico Islands, Autonomous Region of the Azores, Portugal<br>o Reference number: Nota Interna Nº DOC.: 68; Sai-HH/2025/976<br>o Approval dates: 1) 08 September 2025 (CETICA) and 2) 12 September 2025 (BA)<br>o Contact: Maria.FT.Pereira@azores.gov.pt<br>• Ethics committee or Institutional Board: 1) Clinical Ethics Committee (CEC) and 2) Board of Administrators (BA) of Hospital do Divino Espírito Santo de Ponta Delgada, Ponta Delgada, São Miguel Island, Autonomous Region of the Azores, Portugal<br>o Reference number: S-HDES/2025/625<br>o Approval dates: 1) 03 October 2025 (CEC) and 2) 24 October 2025 (BA)<br>o Contact: helio.tm.oliveira@azores.gov.pt
Data sharing statement	A data sharing agreement (joint controller agreement) is in place to allow transfer of data for research collaboration and analysis purposes within the EU PAL-COPD consortium.<br><br>Use of restricted data by third parties will be subject to evaluation by the project coordinators and each participating institution's Principal Investigator, together with relevant legal and ethics, and data protection officers. Data will only be shared upon reasonable request and signing a confidentiality and unilateral data sharing agreement as per the guidance of the local institution's Data Protection Officer.<br>Data access requests should be made to the relevant institution's PI, who will also inform the EU PAL-COPD project coordinator.
Protocol version/Amendment (date)	Version 1.5/Amendment 5 (1 September 2025)<br><br>Replaces:<br>Version 1.4/Amendment 4 (20 June 2025)<br>Version 1.3/Amendment 3 (13 June 2025)<br>Version 1.2/Amendment 2 (10 June 2025)<br>Version 1.1/Amendment 1 (1 April 2025)<br>Version 1/(22 October 2024)
