# Supplementary material for: Integrating palliative care into respiratory care for people with advanced COPD: study protocol for the international EU PAL-COPD stepped wedge cluster randomised controlled trial of the ICLEAR-EU intervention
Source: BMJ Open Respir Res. 2026 Nov 2;13(1):e004150. doi: 10.1136/bmjresp-2026-004150 (PMC13735246; doi:10.1136/bmjresp-2026-004150)
Supplement: Supplementary file 2 [file bmjresp-13-1-s002.pdf]

Supplementary Table 2. Patient and family caregiver demographic data measured at baseline.

**Patient demographics**
– Age
– Sex
– Marital status
– Children
– Cohabitation status
– Hours of transit to hospital where recent hospitalization occurred
– Highest level of education completed
– Employment status
– Comfort of living on household income
– Financial difficulties due to physical condition or treatment
– Country of birth + parent country of birth (if different from respondent birth country)
**Family caregiver demographics**
– Age
– Sex
– Marital status
– Children
– Cohabitation status
– Relationship to the person with COPD
– Living with the person with COPD
– Distance from home of person with COPD (hours)
– Highest level of education completed
– Employment status
– Comfort of living on household income
– Financial difficulties due to physical condition or treatment of family member with COPD
– Country of birth + parent country of birth (if different from respondent birth country)
