# Supplementary material for: Integrating palliative care into respiratory care for people with advanced COPD: study protocol for the international EU PAL-COPD stepped wedge cluster randomised controlled trial of the ICLEAR-EU intervention
Source: BMJ Open Respir Res. 2026 Nov 2;13(1):e004150. doi: 10.1136/bmjresp-2026-004150 (PMC13735246; doi:10.1136/bmjresp-2026-004150)
Supplement: Supplementary file 3 [file bmjresp-13-1-s003.pdf]

Supplementary File 3. RE-AIM outcomes and measures

	Outcome	Measure	Timing of measurement	Completed by
**Reach**	Training attendance	Training attendance list:<br>– Attendance numbers<br>– Professions represented	After each ICLEAR-EU training	Coordinator/ data collector
	ICLEAR-EU Meeting attendance	ICLEAR-EU meeting attendance list:<br>– Attendance numbers<br>– Professions represented	After each ICLEAR-EU meeting	Coordinator/ data collector
	Total number of patients included vs.not included in study	– Admitted for acute exacerbation<br>– Screened for study<br>– Included in study	After every wedge	Coordinator/ data collector
**Effectiveness**	Effectiveness of training	Self-Efficacy regarding end-of-life communication (S-EOLC) (1)<br><br>Palliative and end-of-life care-specific education needs (End-of-life Professional Caregiver Survey (EPCS)) (2)	Pre: 1-4 weeks before training<br><br>Post: 1-4 weeks after start first intervention wedge	ICLEAR-EU team members
	Experiences with ICLEAR-EU	Interview with patients, (bereaved) relatives, and clinicians	Patients: Approx. 4 weeks after hospital discharge<br><br>Bereaved relatives 3 months after bereavement	Local research team

			Clinicians: During follow-up period after last wedge	
**Adoption**	ICLEAR-EU meeting:	Addendum ICLEAR-EU meeting form<br>-How often?<br>-Duration?<br>-How many patients discussed?<br>-How many patients not discussed? Why not?	After each ICLEAR-EU meeting	Coordinator
**Implementation**	Adherence	-Number of inclusions (calculated from inclusion log)<br>-Fidelity checklist (see implementation fidelity)	After every wedge	Coordinator
	Ease of use	Interval scale	After every wedge	ICLEAR-EU team members
	Satisfaction with ICLEAR-EU training/trainer	Evaluation questionnaire	Immediately after training	ICLEAR-EU team members
	Satisfaction with the ICLEAR-EU intervention	Interval scale	After every wedge	ICLEAR-EU team members
	Fidelity	Core components ICLEAR-EU per patient: check based on ICLEAR-EU form or medical record	After every wedge	ICLEAR-EU coordinator for every patient

	Barriers and facilitators to implementation	Short questionnaire with text box<br><br>Regular check-in with local research team by phone	After every intervention wedge	Local research team and coordinator
**Maintenance**	Intention for using ICLEAR-EU in the future	Interval scale	After last wedge	ICLEAR-EU team members, e.g. clinical champion and coordinator
	Organizational intention for long-term implementation	Interval scale	After last wedge	ICLEAR-EU team members, e.g. clinical champion and coordinator
	Experiences with and recommendations for improving usability of service intervention program	Interview with two clinicians from the ICLEAR-EU team	After last wedge	ICLEAR-EU team members, e.g. clinical champion and coordinator

References

1. Mason S, Ellershaw J. Assessing undergraduate palliative care education: validity and reliability of two scales examining perceived efficacy and outcome expectancies in palliative care. Med Educ [Internet]. 2004 Oct 1;38(10):1103–10. Available from: https://doi.org/10.1111/j.1365-2929.2004.01960.x
2. Lazenby M, Ercolano E, Schulman-Green D, McCorkle R. Validity of the End-of-Life Professional Caregiver Survey To Assess for Multidisciplinary Educational Needs. J Palliat Med [Internet]. 2012 Apr 1;15(4):427–31. Available from: https://doi.org/10.1089/jpm.2011.0246
