# Supplementary material for: Integrating palliative care into respiratory care for people with advanced COPD: study protocol for the international EU PAL-COPD stepped wedge cluster randomised controlled trial of the ICLEAR-EU intervention
Source: BMJ Open Respir Res. 2026 Nov 2;13(1):e004150. doi: 10.1136/bmjresp-2026-004150 (PMC13735246; doi:10.1136/bmjresp-2026-004150)
Supplement: Supplementary file 4 [file bmjresp-13-1-s004.pdf]

HORIZON-HLTH-2023-DISEASE-03-01

EU PAL-COPD

Project No. 101136621

Evaluation of the ICLEAR-EU intervention to integrate palliative care in the treatment of people with advanced COPD and their family caregivers: An international stepped wedge cluster RCT in six European countries

Statistical Analysis Plan

Version 2

**Authors**	Julie Stevens (VUB), Kim Beernaert (UGENT), Laure Dombrecht (UGENT), Dries Reynders (UGENT), Marie Van de Walle (Ugent), Elham Nikram (KCL), Charles Normand (KCL) and Peter May (KCL), Mogens Grønvold (UCPH), Nanna Maria Hammer (UCPH)
**Lead participant**	UGent
**Version1**	31 July 2025
**Date version 2**	21 November 2025
**Changes to version 1**	Determined major secondary outcomes, planned subgroup analyses per demographic variable, details of composite scale score calculations.

Main study protocol number: Version 1.5 (01/09/2025)

Trial registration number: ISRCTN45800298

Coordinating/Principal Investigator: Prof. Dr. Koen Pardon, Vrije Universiteit Brussel

Study statistician: Dries Reynders (UGENT)

This SAP is based on guidelines by Gamble, Krishan, & Stocken (2017): Gamble C, Krishan A, Stocken D, et al. Guidelines for the Content of Statistical Analysis Plans in Clinical Trials. *JAMA*. 2017;318(23):2337–2343. doi:10.1001/jama.2017.18556

The current Statistical Analysis Plan (SAP) is provisional, up until data collection is finalized. No major changes are expected. It is subject to revision following a review of the collected data, in accordance with best practices for randomized controlled trials.

Coordinating Investigator:

Prof. Koen Pardon (Vrije Universiteit Brussel)
Prof. Luc Deliens (Vrije Universiteit Brussel)

Principal Investigators:

Vrije Universiteit Brussel
- Prof. Koen Pardon

Lancaster University
- Prof. Nancy Preston

Radboud University Medical Center Medisch Centrum
- Prof. dr. Yvonne Engels

Universiteit Gent
- Prof. Kim Beernaert

Pecsi Tudomanyegyetem - University of Pécs
- Dr. Agnes Csikos

Kobenhavns Universitet
- Prof. Mogens Grønvold

King’s College London
- Dr. Peter May

Universidade Católica Portuguesa
- Prof. Maria Conceição Silva

Fundaçao Gaspar Frutuoso
- Prof. Sandra Martins Pereira

Co-investigators/co-researchers

Vrije Universiteit Brussel
- Prof. Luc Deliens (Co-PI)
- Dr. Julie Stevens (researcher)
- Dr. Frederick Daenen (researcher)
- Ms. Michelle van der Meer (researcher)
- Ms. Mishka Beutels (researcher)

Lancaster University
- Prof. Catherine Walshe (Co-PI)
- Dr. Timothy Gatherall (Pulmonologist)
- Dr. Paul Marsden (Pulmonologist)

- Dr. Didar Karadag Akkaya (Researcher)

Kobenhavns Universitet

- Dr. Nanna Maria Hammer (Researcher)
- Dr. Kristoffer Marsaa (Specialist in pulmonary and palliative medicine)

Universidade Católica Portuguesa

- Diogo Queirós Almeida

Fundaçao Gaspar Frutuoso

- Pablo Hernandez Marrero

Radboud University Medical Center (Nijmegen; NL)

- Dr. Anne Wichmann (Co-PI)
- Manon Wubbels (PhD student)
- Prof. Dr. Kris Vissers (pall care specialist)
- Prof. Dr. Henk Schers (specialised in network primary care)
- Dr Kris Mooren (pulmonologist)

Universiteit Gent

- Dr. Laure Dombrecht (researcher)
- Ms. Marie Van de Walle (researcher)
- Dries Reynders (statistician)

Lung Alliance Netherlands

- Dr Els Verschuur (PI)

Recoverable Signature

X

Signed by: f05ac86b-90b5-4b95-938d-a41f58cb757b

Signature of coordinating investigator

Date: 02-12-2025

Recoverable Signature

X Dries Reynders

Signed by: S-1-12-1-199056507-1153230586-835117958-2170619675/a375ad70-77f7-40a4-b250-a242ac23ce53/

Signature of trial statistician

Date: 02-12-2025

Contents

Partner short names 7

Abbreviations 7

1 Introduction 9

1.1 Background and rationale 9

1.2 Objectives 10

2 Study Methods 11

2.1 Trial Design 11

2.2 Randomization 12

2.3 Sample Size 13

2.4 Framework 13

2.5 Adherence and Protocol Deviations 14

2.6 Interim Analysis and Stopping Guidance 16

2.7 Timing of Final Analysis 17

2.8 Timing of Outcome Assessment 17

3 Trial Population 17

3.1 Screening 17

3.2 Eligibility 18

3.3 Recruitment 19

3.4 Withdrawal/follow-up 20

3.5 Baseline Participant Characteristics 20

4 Statistical Principles 20

4.1 Confidence Intervals and P Values 20

4.2 Analysis Populations 20

4.3 Data Quality 21

5 Analysis 22

5.1 Outcome definitions 22

5.1.1 Demographics 22

5.1.2 Primary outcome 25

5.1.3 Secondary outcomes 25

5.1.4 Health economic evaluation 30

5.1.5 Process evaluation 30

5.2 Analysis methods 30

5.3 Missing data 32

5.4 Additional analyses 32

5.4.1 Economic evaluation 32
5.4.2 Analyses for DSMB reports 33
5.5 Harms 34
5.6 Statistical Software 34
6 References 34
Appendix A: DSMB Report Shell 38

Partner short names

#	Organisation name	Short name
**1**	Vrije Universiteit Brussel	VUB
**2**	Radboud University Medical Centre	RUMC
**3**	Ghent University	UGENT
**4**	University of Copenhagen	UCPH
**5**	Catholic University of Portugal	UCP
**6**	Long Alliantie Nederland	LAN
**7**	European Association for Palliative Care	EAPC
**8**	Lungs Europe	LEU
**9**	accelopment Schweiz AG	accelCH
**10**	European Respiratory Society	ERS
**11**	University of Pécs	UPECS
**12**	University of Lancaster	ULANC
**13**	King's College London	KCL
**14**	Fundaçao Gaspar Frutuoso	FGF

Abbreviations

Abbreviation	Term
ACP	Advance Care Planning
ADRT	Advance Decision to Refuse Treatment
CAT	COPD Assessment Test
COPD	Chronic Obstructive Pulmonary Disease
CSRI	Client Service Receipt Inventory
DSMB	Data and Safety Monitoring Board
EC	European Commission
EPCS	End-of-life Professional Caregiver Survey
EQ-5D-5L	EuroQol 5-Dimension 5-Level
EU	European Union
FEV1	Forced Expiratory Volume in 1 Second
HEU	Horizon Europe

ICLEAR-EU	Intervention to integrate palliative care in the treatment of people with advanced COPD and their family caregivers
ICECAP-SCM	ICEpop CAPability measure for Supportive Care Measure
ICU	Intensive Care Unit
ITT	Intention-To-Treat
MAR	Missing At Random
mMRC	Modified Medical Research Council
MQOL-R	McGill Quality of Life Questionnaire – Revised
PHQ-4	Patient Health Questionnaire-4
PI	Principal Investigator
PRISM-RE-AIM	Practical, Robust Implementation and Sustainability Model – Reach, Effectiveness, Adoption, Implementation, Maintenance
QALY	Quality-Adjusted Life Year
RCT	Randomized Controlled Trial
REDCap	Research Electronic Data Capture
SAP	Statistical Analysis Plan
S-EOLC	Self-Efficacy in End-of-Life Communication
TSC	Trial Steering Committee
VOICES-SF	Views of Informal Carers – Evaluation of Services Short Form
ZBI-12	Zarit Burden Interview – Short Form

1 Introduction

1.1 Background and rationale

Study Rationale

Chronic obstructive pulmonary disease (COPD), a treatable but incurable respiratory condition, is the fourth leading cause of death and the eighth leading cause of poor health worldwide.[1] The global prevalence of COPD is anticipated to increase as populations continue to grow and age.[2] People with COPD experience significant burden from symptoms such as progressively debilitating breathlessness[3] or breathing discomfort, fatigue, anxiety, and depression, with a high co-occurrence of multiple symptoms[4] leading to declining functional status and frequent hospitalizations in the advanced stages of the disease.[3] People with COPD often experience unmet needs, including physical, emotional, social, and existential care needs.[5]

Palliative care can improve the quality of life for patients with COPD by addressing needs across physical, psychological, social, or spiritual domains.[6] However, people with COPD are an underserved population in this regard: although the palliative care needs of people with COPD are as high as those of people with (lung) cancer, referral and access to (specialist) palliative care are limited in comparison.[7–9] If people with COPD do receive palliative care, it is often late into the disease course,[3] such as in the last few weeks of life.[10] Furthermore, if palliative care or end-of-life care is discussed, the frequency and quality of these conversations is generally poor.[11] A proactive approach to palliative care is needed, which integrates palliative care into routine care for COPD; this can reduce the impact of COPD and contribute to patients making choices about their future care through advance care planning (ACP).[12] Integrated palliative care actively involves the patient, family, and multidisciplinary clinical teams who are trained in the palliative care approach, ensuring continuity between all services involved.[13]

The EU PAL-COPD project: integrating palliative care into routine care for people with COPD

The EU PAL-COPD project aims to achieve better quality of life and improved well-being for people with advanced COPD, by integrating palliative care into respiratory care via an innovative, non-pharmacological service-based intervention called ICLEAR-EU. The ICLEAR-EU intervention focuses on early identification of palliative care needs, multidisciplinary care integration including palliative, respiratory, and primary/community care, shared decision-making and advance care planning, and ongoing review. Through this intervention, the EU PAL-COPD consortium aims to reduce unnecessary hospitalizations, mitigate COPD-related impacts, and emphasize a patient-and-family-centred approach to palliative and end-of-life care.

The intervention is based on a model introduced in the United Kingdom (UK), wherein a multidisciplinary team discussed patients with COPD around the time of admission, conducted discussions about ACP and preferred place of death, and communicated information such as medical care plans, ceilings of care, and symptom-specific or palliative care needs to primary care. In this project, this multidisciplinary approach resulted in a reduction in hospital deaths.[14]

From the UK intervention, the consortium adapted the intervention through consultation meetings with clinicians and patients/patient representatives in six countries (Belgium, the Netherlands, the United Kingdom, Denmark, Hungary, and Portugal). After adaptation, the intervention underwent a small-scale pilot-testing in one hospital per country, excluding the hospitals participating in the main RCT, to maximize acceptability and suitability of the intervention for the intended population.

Exacerbations and hospitalisations for COPD are a risk factor for subsequent readmission;[15] data collected from COPD admissions in 13 European countries shows that 35% of patients discharged for an exacerbation were readmitted within 90 days.[16] This leaves patients vulnerable to rapid health deterioration after the first exacerbation and admission to hospital. Early integration of palliative care is essential to reducing potentially preventable readmissions for patients with COPD.[17] In this study, patients with advanced COPD who are admitted to the hospital for more than 48 hours due to an acute exacerbation of their COPD, are invited to participate and will be followed up for 90 days.

1.2 Objectives

The present study aims to compare the ICLEAR-EU intervention to current usual care (treatment as usual) with regard to its:

1. Effectiveness in healthcare systems, as indicated by:

Primary Outcome Measure

a. The percentage of patients who have respiratory-related hospital readmissions within 90 days from baseline, which is counted as the time of signing informed consent (or readmissions registered until death if before 90 days from baseline)

Secondary Outcome Measures

b. **Patient outcomes**: illness perception, quality of life, mental wellbeing, existential wellbeing, presence of advance decisions to refuse treatment and documentation of advance care planning, preferred place of death

c. **Caregiver outcomes**: quality of life, mental wellbeing, existential wellbeing, family carer burden, bereaved caregiver views of quality of care and death

d. **Healthcare utilisation outcomes**: Place of death, concordance between preferred and actual place of death, all-cause mortality, number of readmissions, length of hospital stays on readmission, referrals to specialist palliative care, ICU and emergency department admissions

e. **Cost-effectiveness:** Cost per quality-adjusted life year (QALY)

f. **Process and implementation evaluation:** Feasibility of integration into standard care, barriers and facilitators to implementation, and mechanisms involved in achieving outcomes in each participating country. The analyses of the process and implementation evaluation according to the PRISM-RE-AIM framework are described in further detail in the Process and Implementation Evaluation Protocol.

Major secondary Outcome Measures

Given the large number of secondary outcomes, it is neither statistically nor logistically feasible (time constraints, resource limitations, complexity) to apply the full modelling and sensitivity analysis strategy to each of them. Therefore, we selected a subset of 10 major secondary outcomes based on their clinical relevance, anticipated impact, and alignment with the study objectives. These selected outcomes will be analysed using the same modelling framework and sensitivity analyses as the primary outcome, ensuring methodological consistency and interpretability. This targeted approach allows for robust inference while maintaining analytical feasibility and reducing the risk of multiplicity-related issues. This subset of the selected secondary outcomes includes:

1	Number of readmissions to hospital per patient within 90 days	As extracted from the Electronic Patient Record
2	Length of stay (days) per hospital readmission	As extracted from the Electronic Patient Record
3	Number of times patient attended the emergency department within 90 days	As extracted from the Electronic Patient Record
4	Death in hospital	As extracted from the Electronic Patient Record
5	Patient's illness understanding – Influence of COPD on the patient's life	Brief Illness Perception Questionnaire item: How much does your COPD affect your life?
6	Patient's illness understanding – Patient's concern about their COPD	Brief Illness Perception Questionnaire item: How concerned are you about your COPD?
7	Patient's quality of life	ICECAP-SCM: capability score
8	Mental wellbeing	PHQ-4
9	Existential wellbeing	MQOL
10	Family caregiver burden	ZBI-12

2. Effects on **subgroups**, including subgroups defined by characteristics known to affect health equity and equitable access:
    a. Comparison of outcomes **across participating countries**
    b. Effects on **subgroups** according to gender, age, cohabitation status will be assessed by fitting separate models for each subgroup, rather than within the primary analysis model. In a second step, subgroups according to comfort of living on current income, and hospital characteristics (academic versus general hospital) will be taken into account using separate models.

The overall trial design is described in the full trial protocol. This Statistical Analysis Plan (SAP) provides a detailed description of the methods and statistical analyses for this trial.

2 Study Methods

2.1 Trial Design

A stepped wedge cluster-randomized controlled trial will be used to test the effectiveness of the ICLEAR-EU intervention versus treatment as usual. The trial will proceed in a similar fashion across the six countries. Clustering is at the level of hospital sites, meaning that an entire hospital site will cross over into the intervention at a certain timepoint, wherein all recruited participants will not be individually randomized to either control or intervention. Each country will include three hospitals, each cluster includes one hospital per country. A schematic representation of the trial design is shown in Figure 1.

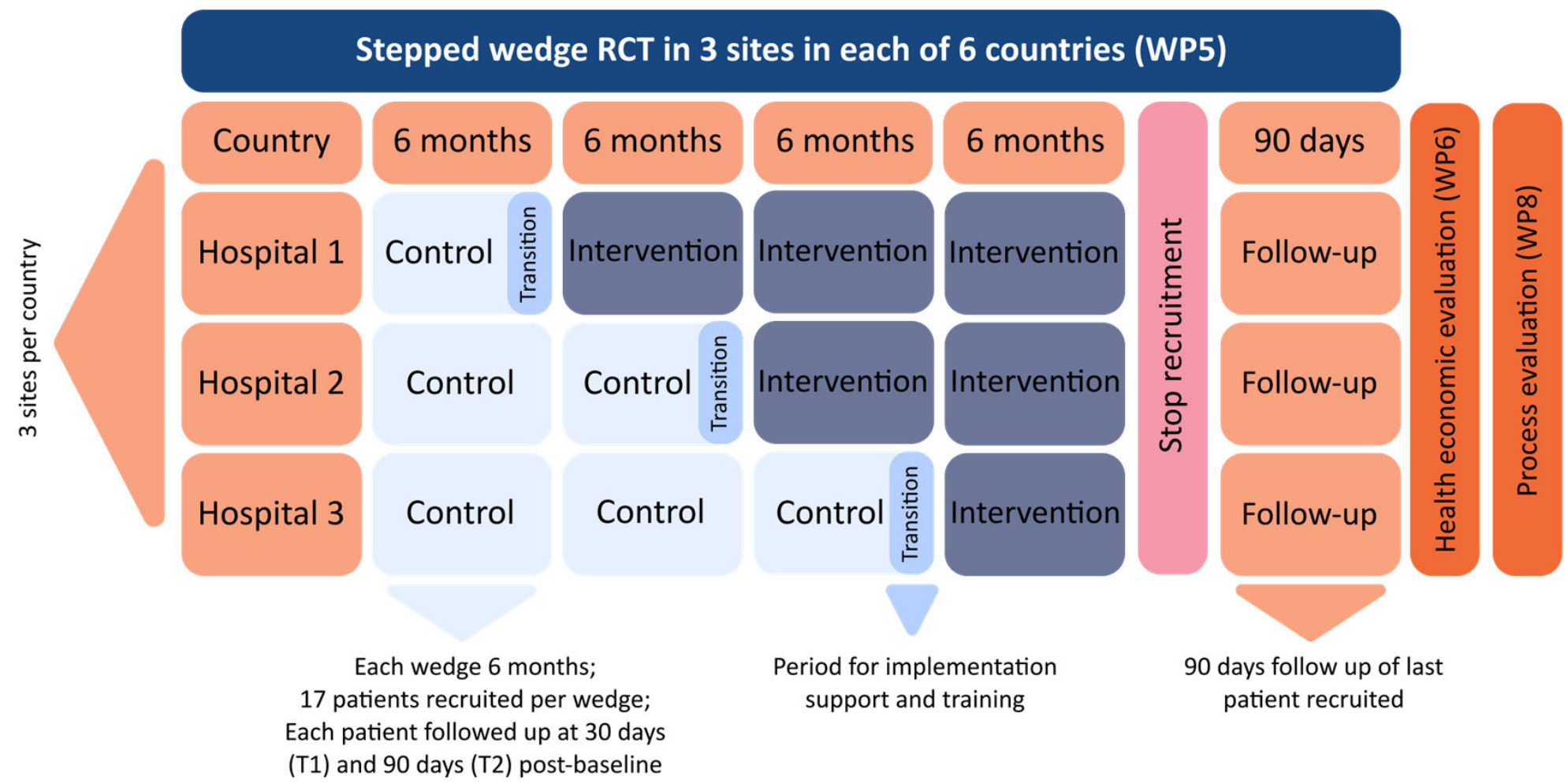

Figure 1. Schematic representation of the stepped wedge RCT

Each hospital will go through four wedges, each wedge with a duration of six months, for a total of 24 months. The timing of cross-over will be randomly assigned at study onset, stratified by country. Country research teams and hospital sites will be notified on whether or not they transition to the intervention wedge 2 months before the start of the new wedge. Before a hospital crosses over from the control condition to the intervention condition, a 30-day transition period will be integrated into the last control wedge, during which clinicians will receive the intervention training and implementation support will be provided. No additional participants will be recruited during this transition period. Control and intervention populations are discrete.

2.2 Randomization

Within each country, three hospitals will be randomized (unweighted) to one of three pre-defined time points for crossover from the control condition (treatment as usual) to the intervention condition: after 6 months, after 12 months, or after 18 months from study start. This ensures that within each country, one hospital transitions to the intervention condition at each of the three steps of the stepped wedge design. Prior to trial initiation (prior to month 1), the trial statistician will generate the randomization sequence using R version 4.3.1 (base-R functions only).

To ensure balanced allocation of study sites across intervention timepoints, a stratified randomisation procedure will be implemented at the hospital level. Stratification will be based on the country of the hospitals, meaning that hospitals will first be grouped by country (the strata), and randomisation will then occur within each group. Hospitals will be randomly assigned to one of three implementation timepoints (IT1, IT2, IT3), with the constraint that each timepoint will include exactly six hospitals, namely one from each participating country. This ensures that each timepoint has equal representation across countries and maintains balance in the rollout of the intervention. The unit of clustering is therefore defined as one hospital per country per timepoint. A fixed random seed will be used to ensure reproducibility of the allocation. Timepoints will be randomly permuted and assigned to hospitals using a simple random sampling approach without replacement. The final allocation will be reviewed manually to confirm that

each country contributes exactly 3 hospitals, each timepoint includes exactly 6 hospitals (one from each country), and the assignment is random and reproducible.

Allocation of the hospitals to the time points will be concealed from both country research teams and hospital staff at onset, until required for implementation. Specifically, the trial statistician will unblind the allocation to the lead institution (VUB) at months 4, 10, and 16. Country research teams will be unblinded by the lead institution at 4, 10, and 16 months to allow preparation of the transition from control to intervention. Each country research team will inform each hospital immediately afterwards whether they will switch from control to intervention at the upcoming crossover point. Implementation preparation and training for the intervention will only take place during the 30-day transition phase. Actual implementation of the intervention will only start on the first day of the first intervention wedge.

Randomization will take place at the end of August, before the start of the trial. The randomization list and the underlying code will be stored on a secure drive hosted by Ghent University, accessible only by the trial statistician. The file will be encrypted and password-protected for the duration of the trial. No changes to the allocation sequence will be permitted after the initiation of the trial.

2.3 Sample Size

The sample size calculation is based on the approach described by Hussey and Hughes.[18] The calculation was done using the R Shiny app developed by Karla Hemming, University of Birmingham[19] and verified in SAS software. We specify a minimum clinically important difference of 15 percent in the number of patients readmitted to hospital within 90 days after baseline. Based on existing literature[15] and expertise of the research team, we estimate that of those recruited at baseline, a proportion of 35% will be readmitted in the future. Similar to our primary outcome measure, this estimate is based on a binary classification, whether a patient was readmitted (yes or no). We use a conservative estimate of the intra-cluster correlation coefficient (ICC) of 0.05 and apply a correction for 30% participant drop-out, such as due to withdrawn consent. This decision was made with the intention of conservatively accounting for potential clustering effects. While the risk of missing data may be lower for the primary outcome, since it can be verified through the electronic medical record (EMR), it remains more relevant for secondary outcomes, as highlighted in recent literature.[20]

Sample size calculations based on these assumptions yield 18 hospitals to be included and randomized across six countries, with three hospitals per country and an average of 17 patients recruited per wedge (68 patients total per hospital, 204 per country, 1224 patients in total). This gives at least 90.0% power to detect a difference of 15 percent at two-sided $\alpha = 0.05$.

We acknowledge that our sample size calculation does not account for potential cluster (i.e., hospital-level) dropout, which could pose a significant risk to the integrity and power of the stepped wedge design. This limitation was discussed within the research consortium and with the Data and Safety Monitoring Board (DSMB). To mitigate this risk, we have included provisions in our contingency plan: we will allow overrecruitment of up to 25 participants per wedge per hospital, and we have identified potential backup hospitals in each participating country that could be activated in the event of cluster dropout.

2.4 Framework

Analysis of the primary outcome are designed to test the effectiveness of ICLEAR-EU over usual care.

Analysis of the secondary outcomes likewise are designed to test the effectiveness of ICLEAR-EU over treatment as usual. Additional **subgroup comparison analyses** are exploratory and intended to:

- identify effects of ICLEAR-EU on different subgroups defined by characteristics known to affect health equity and equitable access (age, gender, socioeconomic status, cohabitation status, and hospital characteristics)
- compare the effects of ICLEAR-EU between countries.

Some data will also be analysed cross-sectionally prior to completion of the trial: E.g., data related to participant demographics will be descriptively analysed at the end of each wedge for the purposes of recruitment reports. The current care practices, assessed via a questionnaire, will be analysed after the first collection in month 6 of the trial, when all participating sites were in a control wedge, to examine characteristics of current palliative care practice without influence of the intervention.

2.5 Adherence and Protocol Deviations

Fidelity to the core components of the intervention will be monitored throughout the trial using the PRISM/RE-AIM framework to evaluate the Reach, Effectiveness, Adoption, Implementation, and Maintenance domains of the intervention alongside key contextual factors. Measurements can be found in Table 1. For more details regarding these measurements, we refer to the process evaluation protocol, available separately.

Table 1. RE-AIM outcomes and measures

	Outcome	Measure	Timing of measurement	Completed by
**Reach**	Training attendance	Training attendance list:<br>Attendance numbers<br>Professions represented	After each ICLEAR-EU training	Coordinator/data collector
	ICLEAR-EU Meeting attendance	ICLEAR-EU meeting attendance list:<br>Attendance numbers<br>Professions represented	After each ICLEAR-EU meeting	Coordinator/ data collector
	Total number of patients included vs.not included in study	Admitted for acute exacerbation<br>Screened for study<br>Included in study	After every wedge	Coordinator/ data collector
**Effectiveness**	Effectiveness of training	Self-Efficacy regarding end-of-life communication (S-EOLC)[26]<br><br>Palliative and end-of-life care-specific education needs (End-of-life Professional Caregiver Survey (EPCS))[27]	Pre: 1-4 weeks before training<br><br>Post: 1-4 weeks after start of the first intervention wedge	ICLEAR-EU team members

	Experiences with ICLEAR-EU	Interview with patients, (bereaved) relatives, and clinicians	Patients: Approx. 4 weeks after hospital discharge<br><br>Bereaved relatives: 3 months after bereavement<br><br>Clinicians: During follow-up period after last wedge	Local research team
**Adoption**	ICLEAR-EU meeting	Addendum ICLEAR-EU meeting form<br>-How often?<br>-Duration?<br>-How many patients discussed?<br>-How many patients not discussed? Why not?	After each ICLEAR-EU meeting	Coordinator
**Implementation**	Adherence	-Number of inclusions (calculated from inclusion log)<br>-Fidelity checklist	After every wedge	Coordinator
	Ease of use	Interval scale	After every wedge	ICLEAR-EU team members
	Satisfaction with ICLEAR-EU training/trainer	Evaluation questionnaire	Immediately after training	ICLEAR-EU team members
	Satisfaction with the ICLEAR-EU intervention	Interval scale	After every wedge	ICLEAR-EU team members
	Fidelity	Core components ICLEAR-EU per patient: check based on ICLEAR-EU form or medical record	After every wedge	ICLEAR-EU coordinator for every patient

	Barriers and facilitators to implementation	Short questionnaire with text box<br><br>Regular check-in with local research team by phone	After every intervention wedge	Local research team and coordinator
**Maintenance**	Intention for using ICLEAR-EU in the future	Interval scale	After last wedge	ICLEAR-EU team members, e.g. clinical champion and coordinator
	Organizational intention for long-term implementation	Interval scale	After last wedge	ICLEAR-EU team members, e.g. clinical champion and coordinator
	Experiences with and recommendations for improving usability of intervention program	- interviews with two clinicians from the ICLEAR-EU team	After last wedge	ICLEAR-EU team members, e.g. clinical champion and coordinator

Deviations from the research protocol will be recorded by trial monitors at the country level and reported to the appropriate parties: project coordinators, ethics committees, and/or the DSMB via the 6-monthly report. Deviations will be reported in the final/main publication.

We consider the following to be deviations from the research protocol:

- Provision of the ICLEAR-EU intervention to patients who were enrolled during the control wedges (see Section 3.3. Analysis population for analyses related to possible contamination effects not falling under direct provision of the intervention to patients enrolled during a control wedge, whose follow-up rolled over into an intervention wedge)
- Enrolment of ineligible participants
- Deviations in timing of intervention rollout which exceed 1 month
- Failure to collect baseline (T0) or follow-up data (T1 and T2) without indicating (reasons for) drop-out

2.6 Interim Analysis and Stopping Guidance

No formal interim analyses are planned, as the trial is considered low-risk. Similarly, no formal stopping rules or guidelines have been prespecified. This approach aligns with recommendations that monitoring intensity should be proportionate to the risk level of the intervention and the trial design.[21]

The trial Data and Safety Monitoring Board (DSMB) will receive 6-monthly reports of recruitment, participant characteristics, and (serious) adverse events. To fulfil their role and make recommendations regarding trial continuation, the DSMB may choose to request additional analyses. The DSMB is responsible for providing recommendations to the Trial Steering Committee (TSC) and the coordinating Principal Investigators (affiliated with the coordinating partner Vrije Universiteit Brussel) about

continuing, modifying, or stopping the trial. As no strict stopping guidelines are in place, the DSMB will make recommendations from their expertise and based on patient safety data from adverse event reports.

2.7 Timing of Final Analysis

The primary outcome of effectiveness will be analysed after trial completion, when all data has been collected. This will be when the T2 data of the last patient of all countries has been collected.

The final data collection via questionnaires will be timed at 90 days (+/- 7 days) after baseline (date of signing informed consent) of the last included patient in the last wedge. Secondary outcomes of effectiveness (see further), as well as the Process Evaluation measures according to the PRISM-RE-AIM framework, will also be analysed after trial completion.

While primary and secondary outcome analyses intended to test the effectiveness of ICLEAR-EU or explore differences in effectiveness between subgroups will be performed after trial completion, some data will also be analysed cross-sectionally prior to completion of the trial (see section 2.4).

2.8 Timing of Outcome Assessment

Questionnaires will be collected from patients and family caregivers at baseline (T0), at 30 days post-baseline (T1), and at 90 days post-baseline (T2). The primary outcome, whether the patient was readmitted to hospital, is measured at 90 days post-baseline (date of signing informed consent) or after death, should this occur before 90 days post-baseline.

For bereaved caregivers, the VOICES-SF questionnaire will be collected at 3 months post-bereavement.

For a more detailed overview of the data collected and its respective timing of assessment, please see Table 4 under section 5.1.3 Secondary Outcomes.

To accommodate scheduling flexibility, a time window of up to 7 days after the scheduled time point is allowed for T0 (baseline), and up to 7 days before or after the scheduled time point for T1 and T2. Questionnaires completed within these windows will be considered valid for the respective time point. Patient and caregiver data will still be included even if data collection falls outside the predefined time windows. Prior to analysis, we will evaluate the distribution of data collection timing and determine which participants' data falls outside the final acceptable time frames for inclusion in the analyses.

3 Trial Population

3.1 Screening

Patients will be screened for eligibility upon admission to the hospital with an acute exacerbation of their COPD. Upon admission, a member of the respiratory team will conduct a first determination of patients' meeting the inclusion criteria.

Screening logs will be maintained in each hospital site to demonstrate how many potential eligible subjects there were, how many were found eligible, how many were approached and how many consented/declined. Reasons why participants declined will be recorded if available. Participants can be rescreened at a later admission to the hospital with an acute exacerbation of their COPD, if at the first timepoint they were deemed ineligible.

3.2 Eligibility

Hospitals: Hospitals that typically admit 100–500 patients annually for COPD-related causes and that indicate a willingness to implement ICLEAR-EU meetings, will be included in the study. Hospital sites will have in-patient respiratory beds.

Hospital and community teams delivering the intervention will vary per country, depending on available services within a given healthcare setting.

Patients and family caregivers: Patients with advanced COPD living at home, who are admitted to the hospital for more than 48 hours (or likely to be admitted for ≥ 48 hours) because of an acute exacerbation of COPD and who will potentially benefit from an integrated palliative care approach, are eligible for participation. Patients may also indicate a family caregiver for participation if they wish. Not including a family caregiver does not exclude the patient from participation.

Inclusion and exclusion criteria for patients and family caregivers are described in Table 2.

Table 2: Patient and family caregiver inclusion and exclusion criteria

Patient	Family Caregiver
**Inclusion criteria**	
• Having a diagnosis of advanced COPD*	• Identified by the patient as the person who gives him or her the most help and support at home on a regular basis
• Admission to the respiratory ward of the hospital that lasts ≥ 48 hours (or likely to be admitted for ≥ 48 hours) for an acute exacerbation	• Age 18 years or over
• Live at home	
**Exclusion criteria**	
• Currently receiving care from a formally recognised specialised palliative care team	• Cognitive impairment preventing informed consent as judged by treating respiratory physician and by the researchers. In case of doubts, the researcher will consult the corresponding treating respiratory physician.
• Cognitive impairment preventing informed consent as judged by treating respiratory physician and by the researchers. In case of doubts, the researcher will consult the corresponding treating respiratory physician.	• Not able to speak or understand the language in which measurements are conducted
• Not able to speak or understand the language in which measurements are conducted, these being:	

- English
- Dutch
- Danish
- Portuguese
- Hungarian

- A patient can be included in the study only once and cannot be re-enrolled during the overall duration study, even if at a different wedge.

***Advanced COPD**

1. Spirometry (FEV1):
   a. Severe COPD: 30% ≤ FEV1 < 50% predicted **OR**
   b. Very severe COPD: FEV1 < 30% predicted

OR

2. High symptom burden:
   a. Modified Medical Research Council (mMRC) > 2 **OR**
   b. COPD Assessment Test (CAT) > 20

OR

3. High-risk exacerbation history:
   a. ≥ 1 exacerbation leading to previous hospitalisation in the past year **OR**
   b. ≥ 1 exacerbation leading to previous ICU admission in the past year

3.3 Recruitment

Recruitment will proceed over the full course of the trial (24 months), so that sufficient participants are recruited per wedge, with the exception of a period of non-recruitment for one month in every hospital in preparation for their transition to the intervention. Over-recruitment up to 25 participants per wedge is allowed to account for this loss. A member of the respiratory team will approach eligible patients concerning study participation, using a standardized introduction text and flyer. Consecutive patients will be recruited as much as possible to avoid recruitment bias. With the patient's agreement, a data collector will check to confirm patient eligibility and invite eligible patients (and the patient's caregiver, if present and consented by the eligible patient) to participate. If the caregiver is not present, a standardized text will be used to ask the patient to identify an eligible caregiver for participation; permission will be sought from the patient to approach this person for participation as soon after the patient's informed consent as possible. Informed consent will be obtained from patients and caregivers after providing information about the purpose of the study and data collection.

3.4 Withdrawal/follow-up

Patients and family caregivers participate voluntarily in the study and are informed of their right to withdraw at any time, for any reason. Withdrawal of consent, withdrawal from follow-up, and loss to follow-up at each time point will be presented within the CONSORT flow diagram when reporting trial analyses. Withdrawal and follow-up will be tabulated by hospital site and whether the withdrawal/ loss to follow-up occurred in the control or intervention condition. The investigators will report, where possible, the reason for withdrawal/loss to follow-up.

3.5 Baseline Participant Characteristics

Table 3 lists participant characteristics (demographics) collected at baseline. Table 4 lists secondary outcomes, including those measured at baseline. We will report demographic information and T0 (baseline) measurements of outcomes for each wedge.

4 Statistical Principles

4.1 Confidence Intervals and P Values

A significance level of 5% will be used in all statistical tests. This implies that statistical tests resulting in p values below 5% have the null hypothesis rejected. 95% confidence intervals will be reported where appropriate. No adjustment for multiple testing will be applied unless explicitly stated.

4.2 Analysis Populations

Analyses will follow the intention-to-treat principle, meaning that participants are analysed according to the treatment condition assigned at the time of their enrolment, regardless of the treatment they actually received. In this stepped-wedge cluster randomized trial:

- Patients enrolled during a **control wedge** will be analysed as part of the control group.
- Patients enrolled during an **intervention wedge** will be analysed as part of the intervention group—even if they receive all, some, or none of the intervention.

Patients readmitted for a COPD exacerbation more than 90 days after enrollment will not be re-enrolled for data collection, meaning that one participant cannot join the study twice (for e.g. first in a control wedge and later in the intervention wedge) even if their follow-up period has ended.

In the stepped-wedge design, patients are assigned to control or intervention conditions based on the timing of their enrolment relative to their cluster's randomized crossover. However, some patients may have outcomes measured after their cluster (hospital site) has transitioned to the intervention phase.

Hospitals are instructed to provide care as usual for patients recruited in the control wedge even if these patients' 90-day follow-up period crosses into an intervention wedge. A **temporal contamination effect** is possible, potentially diluting or misclassifying the exposure effect under the intention-to-treat (ITT) framework. This temporal effect is less likely for the primary outcome measure (hospital readmission yes/no), as contamination would only occur if participants were readmitted after crossover of the hospital site to the intervention wedge. At that time, the binary outcome is already determined, regardless of whether the care they receive during that readmission is influenced by the intervention. Secondary outcome measures such as the number of readmissions, quality of life, or care experiences may be more

susceptible to contamination. These outcomes could be influenced by improved palliative care practices introduced through ICLEAR-EU, even if patients were initially enrolled under control conditions. Therefore, for the primary outcome, and major secondary outcomes (see section 1.2), we will evaluate the robustness of the primary ITT results to potential misclassification of exposure status by carrying out sensitivity analysis by removing all participants with crossover hospital readmissions into an intervention wedge from analysis. After removal of crossover cases, the same statistical model used in the primary ITT analysis will be applied. If the found ITT estimates meaningfully differ from the original results, this may indicate contamination effects or non-adherence to study protocols at crossover. Consistency across models would strengthen confidence in the primary ITT findings.

ITT-population: all patients enrolled, with treatment as assigned (treatment-status of hospital at time of enrolment), regardless of actual received treatment.

Sensitivity-population: a subset of all patients enrolled, excluding patients who were initially recruited during a control wedge but were readmitted after their hospital transitioned into an intervention wedge. We include all patients who were exposed to the same condition (control or intervention) for the entire observation window, and all patients included in a control wedge without readmissions crossing over in an intervention wedge even if their T1 or T2 measures were assessed while their hospital transitioned to an intervention wedge.

4.3 Data Quality

Country research teams responsible for data entry in REDCap received extensive training. Data quality will be periodically checked throughout the trial period. The REDCap system includes automatic flags when information fields are left blank. Text fields in REDCap allow researchers entering data to indicate when questions or instruments were skipped, and to provide the reason for skipping them.

Further, the Data Quality Module in REDCap allows checking for:

- Blank values
- Blank values (required fields only)
- Field validation errors (incorrect data type)
- Field validation errors (out of range)
- Outliers for numerical fields (numbers, integers, sliders, calculated fields)
- Hidden fields that contain values
- Multiple choice fields with invalid values
- Incorrect values for calculated fields
- Fields containing "missing data codes"
- Timely data entry per questionnaire
- Timely completion of the following: patient and family caregiver questionnaires, current care questionnaire, maintenance evaluation form, S-EOLC, EPCS

Members of national teams will conduct routine data verification by comparing source documents (e.g., paper questionnaires) with the corresponding entries in REDCap. This process is intended to identify and correct data entry errors and does not constitute a formal audit. The VUB will randomly check 10% of the data entries per country to evaluate the data quality monthly and flag errors to the national teams for follow-up within one week.

Manual checks will be conducted to ensure timely completion of specific questionnaires that cannot be automatically tracked in REDCap. These include the data retrieval form, the questionnaire for bereaved caregivers, and additional questionnaires completed by ICLEAR-EU team members.

The VUB is responsible for aggregating datasets from each partner country using R (see section 5.6). Together with the raw aggregated dataset, the cleaned "Masterfile", and the working file, a separate syntax of the cleaning, recoding and transformations, and analyses will be documented. Each operation within the syntax will include a comment explaining the rationale for the operation (e.g. inverse coding an item according to questionnaire scoring instructions).

5 Analysis

5.1 Outcome definitions

5.1.1 Demographics

Patient and family caregiver demographic characteristics will be collected at baseline. An overview of the demographic data collected is shown in Table 3. Coding for analysis purposes is mentioned in the table.

Table 3. Patient and family caregiver demographic characteristics to be collected at baseline

**Patient demographics**	
**Data collected**	**Planned grouping for analysis (see further)**
Age: in years [free-text]	Continuous variable
Sex:<br>• Male<br>• Female<br>• Other<br>• Prefer not to answer	Categorical variable: Male, Female, Other/Prefer not to answer (combined if low frequency)
Marital status:<br>• Married or in a relationship<br>• Separated/divorced<br>• Widowed<br>• Single/not in a relationship<br>• Prefer not to answer	Binary variable: In relationship vs Not in relationship.<br>Prefer not to answer reported separately
Children:<br>• I have children under the age of 18<br>• I have children aged 18 years or older<br>• I don’t have any children	Binary variable: Has children vs. No children
Cohabitation status:<br>• With a spouse/partner<br>• With children under the age of 18<br>• With adult children aged 18 years or older<br>• With other persons, please specify: [free-text]<br>• I live alone	Binary variable: Living alone vs. Living with others
Self- reported proximity to the hospital (hours of transit to hospital where recent hospitalization occurred):<br>• Less than ½ hour<br>• ½ - 1 hour<br>• 1-2 hours<br>• More than 2 hours	Categorical variable with 4 categories as collected; may be collapsed depending on distribution
Highest level of education completed:<br>• ISCED 2011 category 0-8 (Country-specific options)	Ordinal variable grouped by ISCED levels: 0-2 (primary, lower secondary)

• Prefer not to answer	3-4 (upper secondary and post-secondary)<br>5-8 (tertiary)<br>Prefer not to answer reported separately
Employment status:<br>• Yes, but I am currently on sick leave (full time or part time)<br>• Yes, but I am currently on care leave (full time or part time)<br>• Yes, I work full time<br>• Yes I work part time. Number of hours: [free-text] hours<br>• No, I am unemployed<br>• No, I am a homemaker<br>• No, I am retired<br>• No I am a student<br>• Other, please specify: [free-text]<br>• Prefer not to answer	Categorical variable with 4 categories:<br>− Employed, currently working<br>− Employed and on sick leave or care leave<br>− Retired<br>− Unemployed/other (student, homemaker)<br>Prefer not to answer reported separately
Comfort of living on household income:<br>• Living comfortably on present income<br>• Coping on present income<br>• Difficult on present income<br>• Very difficult on present income<br>• Don't know<br>• Prefer not to answer	Ordinal variable with 4 categories, 'don't know' and 'prefer not to answer' options reported separately; may be collapsed (e.g., Comfortable vs. Not comfortable)
Financial difficulties due to physical condition or treatment:<br>• Not at all<br>• A little<br>• Quite a bit<br>• Very much<br>• Prefer not to answer	Ordinal variable with 4 categories, 'prefer not to answer' option reported separately, may be collapsed depending on distribution.
Country of birth + parent country of birth (if different from respondent birth country) [Free-text]	Descriptive; may be grouped by region or migration status
**Family caregiver demographics**	
**Data collected**	**Planned grouping for analysis**
Age: in years [free-text]	Continuous variable
Sex:<br>• Male<br>• Female<br>• Other<br>• Prefer not to answer	Categorical variable: Male, Female, Other/Prefer not to answer (combined if low frequency)
Marital status:<br>• Married or in a relationship<br>• Separated/divorced<br>• Widowed<br>• Single/not in a relationship<br>• Prefer not to answer	Binary variable: In relationship vs Not in relationship.<br>Prefer not to answer reported separately
Children:<br>• I have children under the age of 18<br>• I have children aged 18 years or older<br>• I don't have any children	Binary variable: Has children vs. No children
Cohabitation status:<br>• With a spouse/partner<br>• With children under the age of 18	Binary variable: Living alone vs. Living with others

• With adult children aged 18 years or older<br>• With other persons, please specify: [free-text]<br>• I live alone	
Relationship to the person with COPD:<br>• Spouse/partner<br>• Parent<br>• Sister/brother<br>• Daughter/son<br>• Other relative<br>• Friend<br>• Other, please specify: [free-text]	Categorical:<br>Spouse or partner<br>Child<br>Other family<br>Other, not family
Living with the person with COPD:<br>• No<br>• Yes	Binary variable: living together versus living apart
Distance from home of person with COPD (hours):<br>• Less than ½ hour<br>• ½ - 1 hour, 1-2 hours<br>• More than 2 hours	Ordinal variable with 3 categories.
Highest level of education completed:<br>• ISCED 2011 category 0-8 (Country-specific options)<br>• Prefer not to answer	Ordinal variable grouped by ISCED levels: 0-2 (primary, lower secondary)<br>3-4 (upper secondary and post-secondary)<br>5-8 (tertiary)<br>Prefer not to answer reported separately
Employment status:<br>• Yes, but I am currently on sick leave (full time or part time)<br>• Yes, but I am currently on care leave (full time or part time)<br>• Yes, I work full time<br>• Yes I work part time. Number of hours: [free-text] hours<br>• No, I am unemployed<br>• No, I am a homemaker<br>• No, I am retired<br>• No I am a student<br>• Other, please specify: [free-text]<br>• Prefer not to answer	Categorical variable:<br>Employed and currently working<br>Employed and on sick leave or care leave<br>Retired<br>Unemployed/other (student, homemaker)<br>Prefer not to answer reported separately
Comfort of living on household income:<br>• Living comfortably on present income<br>• Coping on present income<br>• Difficult on present income<br>• Very difficult on present income<br>• Don't know<br>• Prefer not to answer	Ordinal variable with 4 categories, 'don't know' and 'prefer not to answer' options reported separately; may be collapsed (e.g., Comfortable vs. Not comfortable)
Financial difficulties due to physical condition or treatment of family member with COPD:<br>• Not at all<br>• A little<br>• Quite a bit<br>• Very much<br>• Prefer not to answer	Ordinal variable with 4 categories, 'prefer not to answer' option reported separately
Country of birth + parent country of birth (if different from respondent birth country) [Free-text]	Descriptive; may be grouped by region or migration status

Nominal or categorical variables will be summarized by absolute (n=) and relative frequencies, with valid percentages in case of missing data. Continuous variables will be summarized using the median and interquartile range (25th–75th percentile), and where relevant, the minimum and maximum values will also be reported. This approach ensures robust descriptive statistics regardless of the underlying distribution. Box plots or histograms can also be drawn for continuous variables to infer visually about the distribution of variables (in some cases the distribution may be very much skewed – e.g. age). Baseline characteristics will be tabulated separately for the intervention and control conditions and inspected for indications of compromised randomization. In addition to overall tables, baseline characteristics will also be presented stratified by country, as the analysis is effectively stratified at the country level. This allows for the identification of within-country imbalances, which are particularly relevant given the potential for country-specific differences in patient populations and care practices. Any clinically meaningful imbalances will be noted and considered in sensitivity analyses or adjusted models, if appropriate.

5.1.2 Primary outcome

The primary outcome is readmission to hospital within 90 days of baseline (or until death if within these 90 days). This will be measured via routinely-collected data in hospital, reported via a data retrieval form. The variable will be treated as dichotomous, indicating whether a patient was readmitted (yes/no).

This endpoint will be evaluated regardless of death ('readmission while alive').

Estimand:

- Population: all patients enrolled in the study
- Treatment: assigned intervention-status of the hospital at which the patient is enrolled, at the time of enrolment
- Outcome: respiratory-related hospital readmissions within 90 days from baseline
- Intercurrent Events:
    - Death: a while-alive strategy. If a patient dies without prior respiratory readmission, the patient will be analysed as not having experienced the primary event
    - All other (e.g. hospital admission due to other reasons): treatment policy – the outcome is evaluated regardless of these intercurrent events. (E.g. while a patient is in hospital for non-respiratory reasons, the patient cannot be readmitted for respiratory-related reasons. This does reflect the potential impact of the planned intervention)
- Summary Measure: log odds ratio for condition (control or intervention) in the GLMM described in section 5.3

5.1.3 Secondary outcomes

The secondary outcomes, along with the corresponding measurement instruments, respondents, and timing of data collection, are described in Table 4. The ten major secondary outcomes (see 1.2) will be analysed using appropriate regression models based on the nature of each outcome (e.g., linear models for continuous outcomes and logistic models for binary outcomes); and sensitivity analyses will follow similar principles as those applied to the primary outcome to ensure methodological consistency and interpretability.

Table 4. *Overview of data collected and timing*

Construct	Data collection measure	Completed by	Timing		
			**T0 (Baseline)**	**T1 (30 days post-baseline)**	**T2 (90 days post-baseline)**

**Patient outcomes**					
Perception of illness	Brief Illness IPQ	Patient	x	x	x
Quality of life	SF-CRQ<br><br>EQ-5D-5L<br><br>ICECAP-SCM	Patient	x	x	x
Mental wellbeing	PHQ-4	Patient	x	x	x
Existential wellbeing	MQOL-R existential subscale	Patient	x	x	x
Preferred place of death + whether this has been discussed with health care professionals	Questionnaire item	Patient	x	x	x
Formal healthcare utilisation and unpaid family caregiving	CSRI	Patient	x	x	x
Presence of advance decisions to refuse treatment (ADRTs) and advance care plans (ACPs)	ICLEAR-EU form and medical notes	Physician/ researcher			x
**[Bereaved] caregiver outcomes**					
	–				
Quality of Life	EQ-5D-5L	Caregiver	x	x	x
Mental wellbeing	PHQ-4	Caregiver	x	x	x
Existential wellbeing	MQOL-R existential subscale	Caregiver	x	x	x
Family carer burden	ZBI-12	Caregiver	x	x	x
Bereaved caregiver views of quality of care and death	VOICES-SF	Bereaved caregiver	3 months post-bereavement		
**Healthcare utilisation of enrolled patients**					
Place of death	Medical notes or phone GP	Researcher	As appropriate		
Concordance between preferred place of death and actual place of death	Questionnaire item Medical notes or phone GP	Researcher	As appropriate		
All-cause mortality	Medical notes	Physician/ researcher	As appropriate		
Number of readmissions to hospital	Medical notes	Physician/ researcher			x

Median length of hospital stays on readmission	Medical notes	Physician/ researcher		x
Number of referrals to specialist palliative care	ICLEAR-EU form and medical notes	Physician/ researcher		x
Intensive Care Unit (ICU) admissions	Medical notes	Physician/ researcher		x
Emergency Department admissions	Medical notes	Physician/ researcher		x

For each (measurement) instrument, the scoring procedures and interpretation guidelines are described below. For multi-item scales, when calculating sum scores, missing items will be replaced by the mean of the items that have been answered if ≤50% of the items are missing. This procedure applies to the following: the Dyspnea, Fatigue, Emotional function, and Mastery domains in the SF-CRQ; the Depression and Anxiety subscales in the PHQ-4 (and/or the total PHQ-4 score); the existential subscale of the MQOL-R; and the ZBI-12. Procedures for handling missing data in single-item scales or in cases where entire multi-item scales are missing, see Section 5.3 on missing data.

Brief Illness Perception Questionnaire (Brief IPQ)

The Brief IPQ is a generic nine-item instrument, designed to assess patients' representations of their illness[22,23]. For the purpose of the EU PAL-COPD study we have included the seven items mentioned below, with each item reflecting a different dimension of illness perception:

- Consequences (i.e., impact of illness)
- Personal Control (i.e., control over illness).
- Treatment Control (i.e., treatment effectiveness)
- Identity (i.e., extent and severity of symptoms)
- Concern (i.e., concern about illness)
- Coherence (i.e., perceived understanding of illness)
- Emotional representation (i.e., emotional effects of illness)

Two items were left out because they were considered upsetting (free-text item on what they believed caused their COPD) or not meaningful (item on expected disease duration) during the pilot test.

All items are scored and analysed individually as single-item scales with scores ranging from 0 to 10. Each item score is treated as a continuous numeric variable (interval scale). For the items 1, 4, 5, and 7 a higher score indicates a more threatening view of the illness, while for items 2, 3, and 6 a lower score indicates a more threatening view of the illness[22,23].

Short-Form Chronic Respiratory Questionnaire (SF-CRQ)

The SF-CRQ is an eight-item instrument designed to measure health-related quality of life in people with COPD exacerbations. The instrument covers four domains, each covered by two individual items:

- Dyspnoea (i.e., while walking on the flat and while sleeping, respectively)
- Fatigue (i.e., feeling worn out and amount of energy, respectively)

- Emotional function (i.e., frustration/impatience and worries/depression, respectively)
- Mastery (i.e., panic due to dyspnoea and control over breathing problems, respectively)

All items are scored on a scale from 1 to 7 (continuous numeric variable on interval scale). Primary analysis for this scale consists of using the four domains, which are scored individually by calculating the mean score within each domain (continuous numeric variable on interval scale). Higher scores implies better quality of life[24].

EuroQoL 5-Dimension 5-level (EQ-5D-5L)

The EQ-5D-5L is a generic instrument for describing and valuing health[25]. This instrument includes five items on health dimensions and one on overall health:

- Items 1–5 (Mobility, Self-Care, Usual Activities, Pain/Discomfort, Anxiety/Depression): Five levels from 1 (no problems) to 5 (extreme problems).
- Item 6 (Visual Analogue Scale): A 0–100 rating of current overall health, with higher scores indicating better health.

For discrepancies between an X-mark and a written number on the paper version of the visual analogue scale, the written number is used.

Responses to Items 1–5 will be used to derive health utility values using the appropriate EuroQol value sets for each participating country. These utility values will be incorporated into the economic evaluation to estimate quality-adjusted life years (QALYs).

The VAS score will not be used in calculating health utility values. However, it will be analysed descriptively as a secondary outcome to assess participants' self-rated health. It will be also explored in relation to utility scores to assess internal consistency of the EQ-5D-5L scores across the sample.

ICECAP-Supportive Care Measure (ICECAP-SCM)

The ICECAP-SCM is a seven-item instrument designed for use in economic evaluations of end-of-life care[26]. This instrument measures capability at the end of life across seven items:

- Choice (i.e., ability to make decisions regarding life and care; 1=never, 4=most of the time)
- Love and affection (i.e., ability to be with people who care; 1=never, 4=most of the time)
- Physical suffering (reverse scored, i.e., experiencing physical discomfort; 1=always, 4=rarely)
- Emotional suffering (reverse scored, i.e., experiencing emotional suffering; 1=always, 4=rarely)
- Dignity (i.e., ability to maintain dignity and self-respect; 1=never, 4=most of the time)
- Being supported (i.e., ability to have needed help and support; 1=never, 4=most of the time)
- Preparation (i.e., opportunity to make wanted preparations; 1=any, 4=most)[26]

All items are scored on a scale from 1 to 4 (with 1 corresponding to lowest level of capability and 4 to the highest level of capability)[26]. A valued capability measure, across the seven items, is derived based on the ICECAP-SCM tariff values, using a designated ICECAP-SCM scoring spreadsheet. The value is anchored on a scale ranging from 0 (representing no capability at the end of life) to 1 (representing full capability at the end of life)[27]. Primary analysis for this scale includes analysing the valued capability measure

(continuous variable), secondary analysis may include looking at individual item scores (categorical variables) due to their conceptual relevance.

Patient Health Questionnaire-4 (PHQ-4)
The PHQ-4 is composed of two ultra-brief screening measures depression (i.e., the PHQ-2), and anxiety (i.e., the GAD-2), respectively. The PHQ-4 consists of four items; two items for each subscale:

- Depression(i.e., Loss of interest/pleasure, and feeling down/depressed)
- Anxiety (i.e., Feeling nervous/anxious, and worrying)

All items are rated on a scale ranging from 0 (not at all) to 3 (nearly every day)[28]. A PHQ-4 total score (0–12) and two subscores (PHQ-2 and GAD-2, each 0–6). Higher scores indicate greater psychological distress[29]. A score ≥ 3 on either subscale indicates potential clinical relevance and the need for further assessment[29]. Primary analysis includes PHQ-4 total score (continuous variable) and both PHQ-2 and GAD-2 scores (continuous variable).

McGill Quality of Life Questionnaire – Revised (MQOL-R): Existential subscale
The MQOL-R measures quality of life of people with life-threatening illnesses across four domains. For the purpose of the EU PAL-COPD study, we will only use the existential sub-scale of the MQOL-R, which consists of 4 items:

1. Purposefulness/meaningfulness
2. Amount of control
3. Felt good about myself as a person
4. Achieving life goals)

All items are scored on a 0–10 scale. Item 2 (i.e., amount of control) is reverse scored (10 - raw score). Only the overall score, which is the mean of all four items after reverse scoring, will be used in analysis (continuous variable), where higher scores reflect better existential quality of life[30].

Zarit Burden Interview – Short Form (ZBI-12)

The ZBI-12 consists of 12 items measuring the perceived impact of informal caregiving. All items are scored on a 0–4 ordinal scale and summed to a total burden score ranging from 0-48 with higher scores reflecting greater caregiver burden[31]. Only the total burden score will be used in analysis (continuous variable).

Self-Developed End-of-Life Questions

The self-developed end-of-life instrument consists of four items measuring patients' preferred place of death, and whether the patients have discussed this with their relatives or members of their care team. These items are descriptive and will be analysed using frequencies, means, and other descriptive summary statistics. No composite scores will be calculated.

VOICES-SF Questionnaire

The VOICES-SF includes 58 items and assesses the type and quality of care received by people at the end-of-life as reported by their bereaved family carers. The items will be analysed descriptively using frequencies, proportions, and mean scores. As the VOICES-SF is a large questionnaire without validated aggregated domain or subdomain scores, we will explore options to derive meaningful composite

measures once data are available, for example through data-driven approaches such as factor analysis or other dimensionality reduction techniques, to enable appropriate aggregation and subsequent analysis.

Healthcare Resource Use

Healthcare resource use will be captured through:

- Routinely collected hospital administrative data on inpatient stays, including number of admissions, total length of stay, and time spent in intensive or high-dependency units.
- An adapted Client Service Receipt Inventory (CSRI) to record formal healthcare utilisation and unpaid family caregiving.

Cost estimation will be performed by combining reported resource use frequencies with nation-specific unit costs, allowing for the calculation of total costs per participant.

5.1.4 Health economic evaluation

We will calculate the incremental cost-effectiveness ratio (ICER), defined as the additional cost per quality-adjusted life year (QALY) gained, by comparing differences in mean costs and QALYs between the intervention and control groups.

5.1.5 Process evaluation

During this trial, we will conduct an embedded process evaluation in all sites. We will use the PRISM-RE-AIM framework to evaluate the Reach, Effectiveness, Adoption, Implementation, and Maintenance domains of the intervention alongside key contextual factors. These will be qualitatively and quantitatively assessed as shown in Table 1. For more details regarding these measurements, we refer to the process evaluation protocol, available separately.

5.2 Analysis methods

All analyses of outcomes will follow intention-to-treat (ITT) unless mentioned otherwise. Participants will be assessed according to whether they entered the study during a control wedge or an intervention wedge.

Primary outcome

To determine the effectiveness of the ICLEAR-EU intervention, we will compare the primary outcome (percentage of patients readmitted for respiratory-related reasons within 90 days, or until death if this occurs before 90 days) between control and intervention participants using a logistic mixed model approach. Effect sizes will be expressed as odds ratios (OR). The model will include a random effect for hospital, and fixed effects for condition (control or intervention), country, and time with additional fixed effects explained below.

Given the stepped-wedge cluster-randomized design, the analysis focuses on within-hospital comparisons over time. A key concern in this context is that within-hospital differences over time may reflect changes in the enrolled patient population or other time-related factors (e.g., seasonal effects), rather than the intervention itself. To address this:

- **Secular trends** (i.e., time trends) that may differ between countries are accounted for by including a **country × time interaction**.
- **Potential confounding due to changes in patient mix over time** is partially addressed by including a set of **individual-level covariates** in the model. While randomization occurs at the cluster (hospital) level, not the patient level, these covariates help adjust for differences in patient characteristics that may vary across time periods within hospitals.

To account for potential confounding, the following predefined individual- and hospital-level covariates will be included to the **primary analysis model** as fixed effects in accordance with prior evidence[16,22] of their association with hospital readmission:

- **Gender (categorical):** Gender differences may influence health-seeking behaviour, disease profiles, and access to care, potentially affecting readmission risk.
- **Age (modeled as a natural spline with 3 degrees of freedom):** Older age is a well-established risk factor for hospital readmission due to increased frailty, comorbidities, and care complexity. To account for potential non-linear effects, age will be included in the model using a natural spline with 3 degrees of freedom.
- **Cohabitation status (binary):** Living alone versus with others may impact post-discharge support and the ability to manage health conditions, influencing readmission likelihood.
- **A fixed-effect interaction between country and time**: This term will allow for varying secular trends (i.e., time trends) between countries.

In a **secondary analysis of the primary outcome**, the following additional covariates and interaction terms will be included to explore their potential influence on the intervention effect:

- **Comfort of living on current income (ordinal):** This variable serves as a proxy for socioeconomic status, which is associated with health outcomes and healthcare utilization.
- **Hospital characteristics (Academic versus general hospital)**: This contextual factor may affect care quality, discharge planning, and follow-up services, thereby influencing readmission rates.

All covariates will be included as fixed effects in the model. Age, the only continuous covariate in the model, will be modeled using a natural spline with 3 degrees of freedom to account for potential non-linearity. Categorical variables will be dummy-coded.

Model assumptions and diagnostics: Model fit and convergence will be evaluated using standard diagnostics (e.g., residual plots, likelihood-based criteria). If convergence issues arise, appropriate steps will be taken to address them.

To evaluate the robustness of the primary ITT results to potential contamination, a sensitivity analysis in the sensitivity-population (see section 5.2) will be carried out by removing all participants with potential for contamination (i.e. removing all participants with readmissions in later wedges after their hospital switched to the intervention). After removal of possibly contaminated cases, the same statistical model used in the primary ITT analysis will be applied.

Secondary outcomes and subgroup comparisons

Secondary outcomes with (at most) one outcome per patient (e.g. length of stay, concordance between preferred and actual place of death) will be analysed using modelling strategies similar to the primary outcome, including a similar approach to sensitivity analysis (at least for the major secondary outcomes as stated in section 1.2), where the appropriate link-function for the generalized linear mixed model will be chosen according to the outcome (logit-link for binary, identity-link for continuous outcomes).

For repeated measures within patients, an appropriate mixed model will be defined, based on the model for the primary outcome. The baseline-measure will be added as predictor, time will be added as categorical predictor, and a treatment-time interaction will be added. Patient-specific random intercepts are added to the model.

We will conduct subgroup and cross-country comparisons of intervention effectiveness per subgroup and country analysed. A fixed effect interaction between condition and country will capture differences in effect sizes between country.

Additional subgroup comparison analyses are exploratory and intended to identify effects of ICLEAR-EU on different subgroups defined by characteristics known to affect health equity and equitable access (age, gender, comfort of living on current income, cohabitation status, and hospital characteristics). Effects on **subgroups** according to gender, age, and cohabitation status will be assessed by fitting separate models for each subgroup, rather than within the primary analysis model. For these subgroup analyses, a treatment × subgroup interaction term will be added as a **fixed effect** to the primary model. In a next step, using the secondary analysis model of the primary outcome, we will assess the effects on subgroups according to comfort of living on current income and hospital characteristics by fitting separate models for each subgroup. For these subgroup analyses, a treatment × subgroup interaction term will be added as a **fixed effect** to the model.

5.3 Missing data

Data not measured because of death are not missing as they are non-existent after death.

As all analyses target a while-alive estimand and the primary endpoint is derived from patient records to which we have access for all enrolled patients, we foresee minimal to no missing data on the primary endpoint (although measurement/misclassification may occur if re-hospitalization in a different hospital was not properly registered).

For the major secondary endpoints, multiple imputation assuming Missing at Random (MAR) will be implemented using MICE with predictive mean matching. The assumption of data MAR is the assumption that the probability of missingness depends only on observed data. This method generates multiple plausible datasets by imputing missing values based on observed relationships between variables, and results from each imputed dataset are pooled. The imputation model will include the primary outcome measure, all ten major secondary outcomes, and baseline covariates (gender, age, cohabitation status, comfort of living on current income, and hospital characteristics). MICE will be run once for all selected endpoints together, as such, one set of imputed datasets will be produced for both the primary and all major secondary outcomes. We will use n = 100 imputations in the MICE procedure. Missing data patterns will be described before imputation using patterns plots, including Permanent Missingness (dropout, no follow-up data after a certain point) and Transient Missingness (certain data points missing, but later reappearance). Missingness will be summarized per group (intervention vs. control), wedge (time point) and key covariates. Both complete-case analyses and imputed analyses will be reported, with the imputed analyses considered primary.

As missingness is likely MNAR,[33,34] additional sensitivity analyses will be performed for the major secondary outcomes where for each endpoint a fixed negative number will be subtracted from the MAR-imputed value (outcome-specific ; to be decided[35]).

5.4 Additional analyses

5.4.1 Economic evaluation

For the health economics analysis in this project, a cost-effectiveness model will be developed with the main objective of combining within-trial cost-effectiveness results with the long-term effects of the intervention, calculated by extrapolating the effects of ICLEAR-EU beyond the follow-up time. Given the chronic nature of COPD, the use of Markov models seems most appropriate.[19]

Prior to conducting primary analysis of cost-effectiveness, we will examine: (i) baseline differences on characteristics associated with outcome and where necessary control for baseline variables in analysis; and (ii) skew, kurtosis and heteroscedasticity in the cost data and fit an appropriate (most likely, nonlinear)

model. We will account for correlated costs and effects using seemingly unrelated regressions, bootstrapping each set of regressions with 1000 replications, and combining these bootstrapped results in estimating cost-effectiveness acceptability curves. Recognizing the uncertainty associated specifically with our trial design, we will employ a stratified two-stage nonparametric bootstrap resampling procedure for clustered data.

In primary analysis, we will take the healthcare system perspective according to the countries represented in the study. In secondary analysis we will additionally incorporate unpaid family care costs to account for potential cost-shifting. For country-specific reporting, we will identify the recommended perspective and other reporting standards (e.g. discount rate, cost-effectiveness threshold) and tailor national reports to these decision-making contexts. As the time horizon of the cost-effectiveness analysis will be the life expectancy of the patient, discounting will be applied at rates recommended by the national guidelines of different participating countries at the time of analysis and checked for sensitivity to this choice. Additional resource use associated with in-patient admissions, length of stay, outpatient attendance and critical care admissions, including information on non-invasive ventilation, invasive ventilation and cardiopulmonary resuscitation, will be estimated either via case report forms or using hospital admissions and discharge databases. Unit costs (starting year of the trial) will be estimated for each country based on published literature and government sources to generate a total cost per trial participant by country and site involved in the trial.

The evidence generated by the stepped wedge cluster randomized trial will be analyzed and used to estimate parameters for the decision analytic economic model. Overall, differences in costs between the pre- and post-intervention periods will be reported, taking consideration of potential seasonal effects within and across clusters. A list of risk factors to be included in a cost regression-based model will be finalized prior to clinical researchers becoming unblinded to economic evaluation results. Quality-of-life data will provide an estimate of the health-related quality of life (HRQoL) weights to be used in the model. The effect of the intervention on mortality and health-related quality of life will be a key input in the model.

Within-trial mortality data will be used to estimate differential mean survival over the period of trial follow-up, adjusting on the basis of EQ-5D-5L data collected during the study. A long-term extrapolation will be undertaken to estimate QALYs over a patient's expected lifetime, considering age, gender, and specific clinical and epidemiological data, as well as national life tables. Probabilistic sensitivity analyses will be conducted to characterize the uncertainty surrounding the ICLEAR-EU intervention adoption decision.

5.4.2 Analyses for DSMB reports

An example unpopulated form for the independent DSMB is included in Appendix A. This form will be used for the six-monthly reports to the DSMB and will be prepared by the trial statistician and data management team of the Universiteit Gent and Vrije Universiteit Brussel. The report will include counts (n=) of patients screened, eligible, and enrolled over the last six-month report period and overall trial duration. Baseline patient characteristics for each DSMB report will be analyzed and reported as described in "Baseline patient characteristics". Since hospitals cross over in sequence, there is a high likelihood of unblinding if the report is stratified by condition (e.g., condition X vs. condition Y). Due to this consideration, the report will not be stratified unless serious concerns regarding efficacy or patient safety arise. Interim outcome analyses (blinded or unblinded) are not formally planned. In cases of serious concerns regarding efficacy or patient safety, the DSMB may also request these analyses.

5.5 Harms

We assume this service-level, non-pharmacological intervention does not present a risk of protocol-related or intervention-related injury and do not anticipate serious adverse events related to the study procedures or the intervention. Adverse events are monitored locally in each country by trial monitors of the respective research team, and will be summarized in the six-monthly report to the DSMB. Adverse events will be addressed via a standard operating procedure (SOP) provided to all sites. Adverse events, if they occur, will be recorded in REDCap. We will not perform any analysis based on adverse events or serious adverse events (S)AEs, as we do not expect our service-level intervention to pose risks significant enough to warrant this. No stopping rules or predefined thresholds for (S)AEs have been established. However, any adverse events or SAEs that occur will be reported descriptively in the final publication if relevant.

5.6 Statistical Software

All statistical analyses, including descriptive analysis and data cleaning, will be conducted using R and Microsoft Excel. The analyses will be performed using the latest stable version of R available at the time of the analysis. R provides a comprehensive suite of statistical and graphical techniques and is widely used in both academic and industry settings for data analysis and reproducible research. Excel will be used for preliminary data exploration, tabulation, and visualization tasks where appropriate, particularly for tasks that benefit from its intuitive interface and spreadsheet functionalities.

All scripts and code used for data cleaning, analysis, and visualization will be documented and version-controlled to ensure transparency and reproducibility. Specific R packages used will be listed in the final report, including their version numbers, to facilitate replication of the results. Any Excel workbooks used in the analysis will also be archived and referenced in the final report to support reproducibility and traceability.

6 References

1. World Health Organization. Chronic obstructive pulmonary disease (COPD). https://www.who.int/news-room/fact-sheets/detail/chronic-obstructive-pulmonary-disease-(copd).

2. Mannino D, Buist A. Global burden of COPD: risk factors, prevalence, and future trends. *Lancet*. 2007;370(9589):765-773. doi:doi:https://doi.org/10.1016/S0140-6736(07)61380-4

3. Iyer A, Sullivan D, Lindell K, Reinke L. The Role of Palliative Care in COPD. *Chest*. 2022;161(5):1250-1262. doi:doi:https://doi.org/10.1016/j.chest.2021.10.032

4. Christensen V, Holm A, Cooper B, Paul S, Miaskowski C, Rustøen T. Differences in Symptom Burden Among Patients With Moderate, Severe, or Very Severe Chronic Obstructive Pulmonary Disease. *J Pain Symptom Manage*. 2016;51(5):849-859. doi:doi:https://doi.org/10.1016/j.jpainsymman.2015.12.324

5. Clari M, Ivziku D, Casciaro R, Matarese M. The Unmet Needs of People with Chronic Obstructive Pulmonary Disease: A Systematic Review of Qualitative Findings. *COPD J Chronic Obstr Pulm Dis*. 2018;15(1):79-88. doi:doi:10.1080/15412555.2017.1417373

6. World Health Organization. Palliative Care Facts Sheet. June 1, 2023. https://www.who.int/europe/news-room/fact-sheets/item/palliative-care

7. Gore J, Brophy C, Greenstone M. How well do we care for patients with end stage chronic obstructive pulmonary disease (COPD)? A comparison of palliative care and quality of life in COPD and lung cancer. *Thorax*. 2000;55(12):1000-1006. doi:doi:10.1136/thorax.55.12.1000

8. Beernaert K, Cohen J, Deliens L, et al. Referral to palliative care in COPD and other chronic diseases: A population-based study. *Respir Med*. 2013;107(11):1731-1739.

9. Rush B, Hertz P, Bond A, McDermid R, Celi L. Use of Palliative Care in Patients With End-Stage COPD and Receiving Home Oxygen: National Trends and Barriers to Care in the United States. *Chest*. 2017;151(1):41-46. doi:doi:https://doi.org/10.1016/j.chest.2016.06.023

10. Bloom C, Slaich B, Morales D, Smeeth L, Stone P, Quint J. Low uptake of palliative care for COPD patients within primary care in the UK. *Eur Respir J*. 2018;51(2). doi:doi:10.1183/13993003.01879-2017

11. Tavares N, Jarrett N, Hunt K, Wilkinson T. Palliative and end-of-life care conversations in COPD: a systematic literature review. *ERJ Open Res*. 2017;3(2). doi:doi:10.1183/23120541.00068-2016

12. Duenk R, Verhagen C, Bronkhorst E, et al. Proactive palliative care for patients with COPD (PROLONG): A pragmatic cluster controlled trial. *Int J COPD*. 2017;12:2795-2806. doi:doi:10.2147/COPD.S141974

13. Gómez-Batiste X, Murray S, Thomas K, et al. Comprehensive and Integrated Palliative Care for People With Advanced Chronic Conditions: An Update From Several European Initiatives and Recommendations for Policy. *J Pain Symptom Manage*. 2017;53(3):509-517. doi:doi:https://doi.org/10.1016/j.jpainsymman.2016.10.361

14. Gudur S, O'Brien F, Salem A, et al. Using Gold Standard Framework Criteria in COPD: Empowering Patients to make Choices about End of Life Care. *Eur Respir J*. 2017;50(Suppl 61):PA4968. doi:doi:10.1183/1393003.congress-2017.PA4968

15. Alqahtani JS, Njoku CM, Bereznicki B, et al. Risk factors for all-cause hospital readmission following exacerbation of COPD: a systematic review and meta-analysis. *Eur Respir Rev*. 2020;29(156):190166. doi:10.1183/16000617.0166-2019

16. Hartl S, Lopez-Campos J, Pozo-Rodriguez F, et al. Risk of death and readmission of hospital- admitted COPD exacerbations: European COPD Audit. *Eur Respir J*. 2016;47(1):113 LP - 121.

17. Kong C, Wilkinson T. Predicting and preventing hospital readmission for exacerbations of COPD. *ERJ Open Res*. 2020;6(2):325-2019.

18. Hussey M, Hughes J. Design and analysis of stepped wedge cluster randomized trials. *Contemp Clin Trials*. 2007;28(2):182-191. doi:doi:https://doi.org/10.1016/j.cct.2006.05.007

19. Hemming K, Kasza J, Hooper R, Forbes A, Taljaard M. A tutorial on sample size calculation for multiple-period cluster randomized parallel, cross-over and stepped-wedge trials using the Shiny CRT Calculator. *Int J Epidemiol*. 2020;49(3):979-995. doi:10.1093/ije/dyz237

20. Schmid-Mohler G, Hübsch C, Braun J, et al. Effect of a nurse-led integrated care intervention on quality of life and rehospitalisation in patients with severe exacerbation of COPD—a pilot study. *Chron Respir Dis*. 2024;21. doi:doi:10.1177/14799731241291067

21. Hohenschurz-Schmidt D, Vase L, Scott W, Annoni M, et al. Recommendations for the development, implementation, and reporting of control interventions in efficacy and mechanistic trials of physical, psychological, and self-management therapies: the CoPPS Statement. *Res Methods Report*. 381:e072108.

22. Broadbent E, Petrie KJ, Main J, Weinman J. The Brief Illness Perception Questionnaire. *J Psychosom Res*. 2006;60(6):631-637. doi:10.1016/j.jpsychores.2005.10.020

23. Broadbent E, Wilkes C, Koschwanez H, Weinman J, Norton S, Petrie KJ. A systematic review and meta-analysis of the Brief Illness Perception Questionnaire. *Psychol Health*. 2015;30(11):1361-1385. doi:10.1080/08870446.2015.1070851

24. Tsai CL, Hodder RV, Page JH, Cydulka RK, Rowe BH, Camargo CA. The Short-Form Chronic Respiratory Disease Questionnaire was a Valid, Reliable, and Responsive Quality-of-Life Instrument in Acute Exacerbations of Chronic Obstructive Pulmonary Disease. *J Clin Epidemiol*. 2008;61(5):489-497. doi:10.1016/j.jclinepi.2007.07.003

25. Herdman M, Gudex C, Lloyd A, et al. Development and preliminary testing of the new five-level version of EQ-5D (EQ-5D-5L). *Qual Life Res*. 2011;20(10):1727-1736. doi:10.1007/s11136-011-9903-x

26. Sutton EJ, Coast J. Development of a supportive care measure for economic evaluation of end-of-life care using qualitative methods. *Palliat Med*. 2014;28(2):151-157. doi:10.1177/0269216313489368

27. Huynh E, Coast J, Rose J, Kinghorn P, Flynn T. Values for the ICECAP-Supportive Care Measure (ICECAP-SCM) for use in economic evaluation at end of life. *Soc Sci Med*. 2017;189:114-128. doi:10.1016/j.socscimed.2017.07.012

28. Kroenke K, Spitzer RL, Williams JBW, Löwe B. An Ultra-Brief Screening Scale for Anxiety and Depression: The PHQ–4. *Psychosomatics*. 2009;50(6):613-621. doi:10.1016/S0033-3182(09)70864-3

29. INSTRUCTION MANUAL - Instructions for Patient Health Questionnaire (PHQ) and GAD-7 Measures. Accessed. Accessed August 12, 2025. https://www.phqscreeners.com/images/sites/g/files/g10016261/f/201412/instructions.pdf

30. Cohen S. *McGILL QUALITY OF LIFE QUESTIONNAIRE-Revised, Expanded, and Original Versions*.

31. Bédard M, Molloy DW, Squire L, Dubois S, Lever JA, O'Donnell M. The Zarit Burden Interview: A New Short Version and Screening Version. *The Gerontologist*. 2001;41(5):652-657.

32. DH / NHS Medical Directorate / End of Life Care. First national VOICES survey of bereaved people: key findings report. Published online 2012. Accessed March 25, 2024. ttps://assets.publishing.service.gov.uk/media/5a7ccc42ed915d63cc65ce79/First-national-VOICES-survey-of-bereaved-people-key-findings-report-final.pdf

33. Preston N, Fayers P, Walters S, Pilling M, Grande G, Et al. Recommendations for managing missing data, attrition and response shift in palliative and end-of-life care research: part of the MORECare research method guidance on statistical issues. *Palliat Med*. 2013;27(10):899-907.

34. Higginson I, Evans C, Grande G, Preston N, Morgan M, Et al. Evaluating complex interventions in end of life care: the MORECare statement on good practice generated by a synthesis of transparent expert consultations and systematic reviews. *BMC Med*. 2013;11:1-11.

35. Cro S, Morris TP, Kenward MG, Carpenter JR. Sensitivity analysis for clinical trials with missing continuous outcome data using controlled multiple imputation: A practical guide. *Stat Med*. 2020;39(21):2815-2842. doi:10.1002/sim.8569

Appendix A: DSMB Report Shell

A stepped wedge randomized controlled trial and process evaluation of the ICLEAR-EU intervention to integrate palliative care in the treatment of people with advanced COPD and their family caregivers: Report to the independent Data and Safety Monitoring Board

Administrative information

Study title: A stepped wedge randomized controlled trial and process evaluation of the ICLEAR-EU intervention to integrate palliative care in the treatment of people with advanced COPD and their family caregivers

Main study protocol number:

Trial registration number: https://www.isrctn.com/ISRCTN45800298

Protocol version corresponding to this DSMB report:

Date of protocol version corresponding to this DSMB report:

Coordinating/Principal Investigator: Prof. Dr. Koen Pardon, Vrije Universiteit Brussel

Study statistician:

Date of report:

Cutoff date for data used in this report:

Last DSMB meeting date:

Report prepared by:

Executive Summary

**Report Overview**	
**Enrollment Status**	<State the number of participants screened and the number enrolled. Mention here any discrepancies between target and actual enrolment per wedge.>
**Subject Status**	<Mention here the number of participants with a baseline measurement, the number with T1 measurement, and the number with T2 measurement. State the number of participants withdrawn from the study.>
**Stopping guidelines**	<The trial does not use formal stopping guidelines.<br>However, if concerns for participant safety are raised which are so significant that they possibly indicate stopping the trial, they should be mentioned here.>
**Adverse events**	<Mention here the number of adverse events and serious adverse events>
**Protocol Deviations**	<Mention here any deviations from the most recent version of the study protocol>
**Trial monitoring**	<Site visits, fidelity monitoring, …>

Synopsis

**Study title**	A stepped wedge randomized controlled trial and process evaluation of the ICLEAR-EU intervention to integrate palliative care in the treatment of people with advanced COPD and their family caregivers
**Principal investigator (per country)**	
**Study sites (per country)**	
**Start date of each site**	
**Planned enrollment (past 6 months)**	17 Patients
**Study design**	Stepped wedge cluster-randomized controlled trial: randomisation at the hospital level
**Study objectives**	**1. Effectiveness evaluation:** To compare the ICLEAR-EU intervention to current usual care regarding its effectiveness in healthcare systems, cost-effectiveness, effects on subgroups known to affect health equity, and effectiveness/cost-effectiveness in different healthcare systems across Europe.<br><br>2. **Process evaluation**: To evaluate the implementation processes of the ICLEAR-EU intervention and the feasibility of its integration into usual care across European settings, the contextual barriers and facilitators for effective and sustainable implementation, and the mechanisms involved in reaching the outcomes in each country as perceived by patients, family caregivers, and care providers.
**Intervention**	ICLEAR-EU: Non-pharmacological, service-based intervention integrating palliative care in respiratory care for people with advanced COPD
**Inclusion Criteria**	**Patients**<br>• Having a diagnosis of advanced COPD:<br>1. Spirometry (FEV1):<br>a. Severe COPD: 30% ≤ FEV1 < 50% predicted OR

	b. Very severe COPD: FEV1 < 30% predicted<br>2. High symptom burden:<br>a. Modified Medical Research Council (mMRC) mMRC > 2 OR<br>b. COPD Assessment Test (CAT) CAT > 20<br>3. High risk exacerbation history:<br>a. ≥ 1 exacerbation leading to previous hospitalization in the past year OR<br>b. ≥ 1 exacerbation leading to previous ICU admission in the past year<br>• Admission to the respiratory ward of the hospital that lasts ≥ 48 hours (or likely to be admitted for ≥ 48 hours) for an acute exacerbation<br>• Living at home<br>Family caregivers<br>• Identified by the patient as the person who gives him or her the most help and support at home on a regular basis<br>• Age 18 years or over
**Exclusion Criteria**	**Patients**<br>• Currently receiving care from a formally recognized specialized palliative care team<br>• Cognitive impairment preventing informed consent<br>• Not speaking or understanding the language in which measurements are conducted<br>• Patient included during a control wedge cannot be enrolled again for measurement in an intervention wedge<br>**Family caregivers**<br>• Cognitive impairment preventing informed consent<br>• Not speaking or understanding the language in which measurements are conducted

**Study Outcomes**	Primary endpoint:<br>• Percentage of patients who are readmitted to hospital within 90 days of baseline (or until death, if within these 90 days).<br><br>Secondary endpoints at 30 days (T1) and 90 days (T2) post-baseline:<br>**Patients**<br>• Perception of illness<br>• Quality of life<br>• Mental wellbeing<br>• Existential wellbeing<br>• Preferred place of death<br>• Presence of advance decisions to refuse treatment and documentation of advance care planning decisions<br><br>**Family caregivers**<br>• Quality of life<br>• Mental wellbeing<br>• Existential wellbeing<br>• Family carer burden<br>• Views on quality of care and death (bereaved caregivers only)<br><br>**Healthcare utilisation of enrolled patients**<br>• Place of death<br>• Concordance between preferred and actual place of death<br>• All-cause mortality<br>• Number of readmissions<br>• Median length of hospital stay on readmission<br>• Number of hospital days<br>• Number of referrals to specialist palliative care<br>• Number of intensive care unit (ICU) admissions<br>• Number of emergency department admissions<br><br>**Cost-effectiveness**<br>• Patient quality-adjusted life years (primary endpoint)<br><br>**Process evaluation**<br>• PRISM/RE-AIM (context, reach, effectiveness, adoption, implementation and maintenance)
**Study stopping guidelines**	None pre-specified.

1. Report overview

[Summary of the report with action points for the DSMB]

2. Response to recommendations from most recent prior DSMB meeting

**Date of most recent prior DSMB meeting**
**Recommendation 1 by DSMB**
**Response to recommendation 1 by the RCT partners and/or consortium**
**Re-evaluation necessary?**
**Recommendation 2 by DSMB**
etc.

Open session report

3. Enrollment status

Table 1 shows the enrollment status of patients and family caregivers in each hospital study site and per country. The status is shown for enrollment within the past 6 months, and enrollment over the complete trial duration to the date of the report, which is X months.

Table 1. Participant enrollment status

	**Study site**					
	**Hospital 1**		**Hospital 2**		**Hospital 3**	
	Patients (N)	*Caregivers (N)*	*Patients (N)*	*Caregivers (N)*	*Patients (N)*	*Caregivers (N)*
**Country 1**						
**Past 6 months:**						
Screened						
Eligible						
Enrolled						
Baseline questionnaire completed						

**Complete trial duration (X months)**						
Screened						
Eligible						
Enrolled						
Baseline questionnaire completed						
**Country 2**						
**Past 6 months**						
Screened						
Eligible						
Enrolled						
etc.						

4. Participant status

Table 2 shows the status of participants in each hospital study site per country, over the past 6 months and for the duration of the trial, which is currently X months. Currently, X patients have been evaluated for the primary outcome (readmission to hospital for a respiratory exacerbation within 90 days, or until death if sooner than 90 days). X patients and X family caregivers have completed T1 measurement, 30 days post-baseline. X patients and X family caregivers have completed T2 measurement, 90 days post-baseline.

X patients and X family caregivers have withdrawn from the study, X patients and X family caregivers have been lost to follow-up; reason are listed in Table X.

Table 2. Participant status

	**Study site**					
	**Hospital 1**		**Hospital 2**		**Hospital 3**	
	Patients (N)	*Caregivers (N)*	*Patients (N)*	*Caregivers (N)*	*Patients (N)*	*Caregivers (N)*
**Country 1**						
**Past 6 months:**						

Primary outcome data collected (Patients)						
T1 (30 days) data collected						
T2 (90 days) data collected						
Withdrew						
Reasons						
Lost to follow-up						
Reasons						
**Complete trial duration (X months)**						
Primary outcome data collected (Patients)						
T1 (30 days) data collected						
T2 (90 days) data collected						
Withdrew						
Reasons						
Lost to follow-up						
Reasons						
**Country 2**						
**Past 6 months**						
Primary outcome data collected (Patients)						
T1 (30 days) data collected						
T2 (90 days) data collected						

etc,						

II. Closed session report

5. Baseline characteristics

[These should be summary and not per-participant]

Table 3 provides an overview of participant characteristics per hospital per country.

Table 3. Participant baseline characteristics

	**Study site**					
	**Hospital 1**		**Hospital 2**		**Hospital 3**	
	Patients (N)(%)[a]	*Caregivers (N)(%)*	*Patients (N)(%)*	*Caregivers (N)(%)*	*Patients (N)(%)*	*Caregivers (N)(%)*
**Country 1**						
Gender						
Female						
Age (Mean, Standard deviation)						
etc.						
**Country 2**						
etc,						

a. Percentages are given as % of total enrolled population

6. Participant safety

Important information regarding safety reporting (from Adverse Event Reporting SOP):

The investigators do not anticipate serious adverse events arising from the intervention or study procedures. However, clinical deterioration and death are possible in this population of patients with advanced COPD. Therefore, deaths, hospitalisations, life threatening or medically significant/important events must be reported as serious adverse events only if they are (potentially) related to the intervention or study procedures. Deaths and hospitalisations not related to the intervention or study procedures will be recorded as part of the study data collection, but not reported as serious adverse events.

Distress should be considered serious if it is unresolved (e.g. cannot be mitigated with the distress protocol) or includes thoughts of self-harm, or thoughts or intent of suicide.

Adverse events

Adverse events and serious adverse events are shown in Table X. Within the past 6 months [or after the last DSMB meeting if less than 6 months, insert date], XX serious adverse events (SAE) occurred. Details of SAE are reported in [[Appendix], if applicable [if SAEs occur, summarize them in appendix].

Table X. (Serious) adverse events

	**Study site**					
	**Hospital 1**		**Hospital 2**		**Hospital 3**	
	Patients (N)(%)	*Caregivers (N)(%)*	*Patients (N)(%)*	*Caregivers (N)(%)*	*Patients (N)(%)*	*Caregivers (N)(%)*
Country 1						
**Past 6 months**						
Adverse events (AE)						
Total number of AE						
Number of participants who experienced at least 1 AE						
Serious adverse events (SAE)						
Total number of SAE						
Number of participants who experienced at least 1 SAE						
etc,						
**Complete trial duration (X months)**						
Adverse events (AE)						
Total number of AE						
Number of participants who experienced at least 1 AE						

Serious adverse events (SAE)						
Total number of SAE						
Number of participants who experienced at least 1 SAE						
etc,						
Country 2						
**Past 6 months**						
etc,						

7. Protocol deviations

Serious breaches

These are serious breaches of the protocol or good GCP guidelines which might significantly affect the safety of trial participants or jeopardize the reliability of the data collected. Reports are for the past 6 months or since last DSMB meeting.

A detailed description of serious breaches is reported in [Appendix, if applicable]

Table X. Serious breaches

**Country 1**						
**Summary description of breach**						
**Date confirmed**						
**Action taken**						
[The above three headings can be repeated however many times is necessary]						
**Country 2**						
**Description of breach**						
etc,						

Protocol deviations and amendments

These are deviations from, and amendments to, the protocol version linked to this DSMB report + the protocol version linked to the previous DSMB report (if amendments have been made).

Changes to study procedures which are rolled out after amendments are agreed on by the consortium and approved by the relevant ethics committee(s) should not be reported as deviations, but rather as amendments.

A detailed description of protocol deviations is reported in [Appendix, if applicable].

Table X. Protocol deviations

**Country 1**	
**Description of deviation**	
**Date confirmed**	
**Action taken**	
[The above three headings can be repeated however many times is necessary]	
**Protocol amendment information**	
Amendment number	
Description of amendment	
Date of approval by project Supervisory Board	
Date of submission to local ethics committee	
Date of approval by local ethics committee	
[Headings for protocol amendment can be repeated however many times necessary]	
**Country 2**	
etc.	

8. Trial monitoring

[Reports of six-monthly trial monitoring per country (see Trial Management and Monitoring Plan): please attach the report per country to this DSMB report.]

9. Interim outcome data

[The sample size does NOT account for interim analysis. This heading should only be included if explicit requests have been made from the DSMB to assess a serious safety concern or other serious concern regarding the trial.]

Appendices to the DSMB Report

Appendices may include but are not limited to:

- Output from data analysis software (SPSS, R, SAS, …)
- Additional figures or tables as required.
- Details of Serious Adverse Events related to study procedures or intervention: attach SAE reporting forms for each SAE since last DSMB report.
- Additional details of protocol deviations or serious breaches if required.

Please include separate appendices for the open and closed sessions.

EU PAL-COPD is funded by the European Union. Views and opinions expressed are however those of the author(s) only and do not necessarily reflect those of the European Union or the European Health and Digital Executive Agency (HaDEA). Neither the European Union nor the granting authority can be held responsible for them [grant number 101136621]. This project is also supported by the UK Research and Innovation (UKRI) [grant numbers 10109731 and 10109782], the Ministry of Culture and Innovation of Hungary from the National Research, Development and Innovation Fund [grant number 2020-2.1.1-ED-2023-00260], and the Swiss State Secretariat for Education, Research and Innovation (SERI).
