# Supplementary material for: Integrating palliative care into respiratory care for people with advanced COPD: study protocol for the international EU PAL-COPD stepped wedge cluster randomised controlled trial of the ICLEAR-EU intervention
Source: BMJ Open Respir Res. 2026 Nov 2;13(1):e004150. doi: 10.1136/bmjresp-2026-004150 (PMC13735246; doi:10.1136/bmjresp-2026-004150)
Supplement: Supplementary file 5 [file bmjresp-13-1-s005.pdf]

Supplementary Table 3. Comparison between indirect effects of being hospitalised during an intervention wedge and receiving the ICLEAR-EU intervention.

Indirect effects of being hospitalised during an intervention wedge	Receiving the ICLEAR-EU intervention
Professionals learn how to identify palliative care needs	A specific tool (ESAS-r or a locally utilized assessment tool alongside a standard clinical assessment) will be used for this
Professionals discuss “their” patients’ treatment and care plan during their regular and usual team meetings	Specific roles will be attributed within the ICLEAR-EU team<br><br>Professionals from specific clinical specialties will be invited to participate in the ICLEAR-EU meetings<br><br>Patients (who consented for participation) will be discussed using the standardized approach established in the EU PAL-COPD Intervention Manual
Professionals will improve their communication competencies (this might or not include communication about goals of care, agreement on levels of escalation, and ACP conversations)	Communication about goals of care, agreement on levels of escalation, and ACP conversations will happen with the patient (and family, if appropriate)
Professionals will document or not the summary of the content of their communication about goals of care, agreement on levels escalation, and ACP conversations in the existing healthcare record. This might or not be shared	The ICLEAR-EU form will be completed and shared with the patient and healthcare team in both hospital and community settings
Ongoing review of treatment plan according to usual practices	Ongoing review of treatment plan using a standardized approach as defined in the ICLEAR-EU manual
